# Design strategies for composite matrix and multifunctional polymeric scaffolds with enhanced bioactivity for bone tissue engineering

**DOI:** 10.3389/fchem.2022.1051678

**Published:** 2022-11-28

**Authors:** Shikha Kumari, Soumya Katiyar, Darshna, Aditya Anand, Divakar Singh, Bhisham Narayan Singh, Sarada Prasanna Mallick, Abha Mishra, Pradeep Srivastava

**Affiliations:** ^1^ School of Biochemical Engineering, IIT BHU, Varanasi, India; ^2^ Department of Ageing Research, Manipal School of Life Sciences, Manipal Academy of Higher Education, Manipal, Karnataka, India; ^3^ Department of Biotechnology, Koneru Lakshmaiah Education Foundation, Vaddeswaram, Andhra Pradesh, India

**Keywords:** scaffold, bone tissue engineering, bioactivity, regenerative medicine, composite matrix

## Abstract

Over the past few decades, various bioactive material-based scaffolds were investigated and researchers across the globe are actively involved in establishing a potential state-of-the-art for bone tissue engineering applications, wherein several disciplines like clinical medicine, materials science, and biotechnology are involved. The present review article’s main aim is to focus on repairing and restoring bone tissue defects by enhancing the bioactivity of fabricated bone tissue scaffolds and providing a suitable microenvironment for the bone cells to fasten the healing process. It deals with the various surface modification strategies and smart composite materials development that are involved in the treatment of bone tissue defects. Orthopaedic researchers and clinicians constantly focus on developing strategies that can naturally imitate not only the bone tissue architecture but also its functional properties to modulate cellular behaviour to facilitate bridging, callus formation and osteogenesis at critical bone defects. This review summarizes the currently available polymeric composite matrices and the methods to improve their bioactivity for bone tissue regeneration effectively.

## 1 Introduction

Tissue engineering makes an attempt to recover, retain, strengthen, or significantly boost tissue functionality when ailment or trauma hampers it. To accomplish the above, novel biological alternative substitutes must be established (Baptista and Guedes, 2021). Even though bone tissue may quickly regenerate, when disease, injury, or infection impairs this ability, new approaches are needed to aid bone regeneration (Amini et al., 2012). Bone tissue engineering focuses on methods to repair damaged bone by creating substitute structures that can perform the protective and structural functions of healthy bone while sustaining stresses placed on the human body from inside and outside (Amini et al., 2012). For bone healing to occur, the generated BTE matrix must simultaneously promote cell growth and the synthesis of new bone tissue (Amini et al., 2012). The most often transplanted human tissue, after blood, is bone (Shegarfi and Reikeras, 2009). Therefore, bone autografts, in which a portion of a patient’s normal bone tissue is surgically removed and relocated to the afflicted region, are still the gold standard approach for BTE. Although this operation is simple to perform, it has drawbacks such as a limited supply, a risk of rejection, and morbidities at the donor site (Nuss and von Rechenberg, 2008). Researchers have created new approaches to address these issues, including biocompatible permanent metal implants. However, some individuals are at risk for rejection, necessitating additional surgical procedures to remove these implants. Additionally, they have incompatible mechanical characteristics that might cause stress shielding and fracture or fatigue failure modes (Nuss and von Rechenberg, 2008).

However, ceramic implants can be used temporarily and permanently for such conditions. Yet, polymeric and composite materials have recently been the focus of research and development (Skorulska et al., 2021). Almost all polymeric materials and their composites are biologically compatible, bio-resorbable, and also have Young’s modulus interoperable with bone. It implies that once transplanted, they will promote the bone regeneration process and gradually be overtaken by newly formed tissue, leaving no scars once the process of bone healing is complete (Roseti et al., 2017a). Some other alternative choice is ceramic and bio-polymeric composites, which combines the functions of a polymer network reinforced with biologically active ceramic particles. These materials outperform others in terms of performance and mechanical behaviour, including osteoconductive effects (Wang et al., 2017). The scaffold (Do et al., 2015; Wang et al., 2020), a three-dimensional biological or synthetic artificial structure used to support bone compressive damages and allow bone tissue repair and re-growth (Roseti et al., 2017a; Wang et al., 2017), is the principal temporary treatment for BTE. For BTE to be successfully applied, four key scaffold characteristics must be met: biological, structural, related material composition, and regarding production procedures. The resultant materials have outstanding osteoinductivity, osteoconductivity, and biocompatibility as potential benefits, offering a promising new technique for bone healing. Scaffold material composition is essential in creating artificial bone because they serve as the physical foundation for artificial grafts (Noori et al., 2017). The ideal and effective scaffold material should include attributes comparable to native bone, ensuring favourable physiochemical surroundings and biomechanical assistance for seed cell attachment, migration, multiplication, osteogenic differentiation potential, and neo-angiogenesis on the scaffold framework.

Last but not least, it must permit gradual integration into the host tissue throughout the healing process to support regular loads (Mishra et al., 2016; Roseti et al., 2017b). To be organically digested, scaffold degradation products also need to be non-toxic and non-immunogenic. Mesenchymal stem cells homing, osteoblast differentiation, extracellular matri and osteoid mineralization, and the development of terminally differentiated osteocytes all play significant roles in bone formation during the regeneration process (Wang et al., 2013a).

Several materials are being utilized to fabricate bone tissue scaffolds, including both natural and synthetic polymers. Researchers incorporate various bioactive molecules, inorganic materials like hydroxyapatite, bioactive glass, and metallic and non-metallic nanoparticles to enhance these scaffolds’ bioactivity. Since inorganic materials bioglass possesses elastic modulus similar to the cortical bone, they are widely used in the fabrication process. Whereas natural polymers possess less than 70 MPa compressive strength, and synthetic polymers exhibit about 10 GPa (Gunatillake et al., 2003; Pilipchuk et al., 2015; Chocholata et al., 2019). Therefore, they are combined to form a replicating matrix for bone tissue. With the help of bioactive scaffolds, the delivery of drugs and growth factors has been enhanced, which helps in the overall bone healing process and improves bone regeneration.

Bioactivity of the scaffolds is the ability to influence the scaffold’s biological surroundings. Enhancing the capacity for the formation of apatite layer, differentiation of osteoblast cells, and the formation of bone ECM falls under this category. Because of their superior bioactivity and ability to form strong bonds with the host bone tissue, modern bioceramics including hydroxyapatite, tricalcium phosphate, composite of HAp/TCP, and 45S5^®^ bioglass are broadly employed as bone biomaterials. Their comparatively poor mechanical strength, especially their lower toughness value, which restricts their application to just low-load bearing parts of the human body, is these bioceramics’ principal drawback. Understanding the attachment of ceramics to living bone and methods for testing the bonding potential is crucial for the development of neo-bioactive ceramics for load-bearing based bone tissue restoration (Wu and Xiao, 2009a).

Recently researchers have started exploring the potential of 3D scaffolds for tissue regeneration. They have developed a number of applications for these scaffolds, with researchers hypothesising that these 3D scaffolds will possess high structural stability and they will also provide a 3D microenvironment for tissue regeneration, mimicking the functionality of natural tissue. When these scaffolds are implanted, they create a local bioactive environment that helps the injured or missing tissue to recover. Synthetic and natural polymers have been widely used as biomaterials to create these scaffolds, in large part because of their enormous diversity (Stratton et al., 2016).

The natural materials include polysaccharides like chitosan, hyaluronic acid, gelatin, starch, etc. Likewise, collagen, fibrin gels, silk fibroin, etc., also help cell adhesion. The only limitation is their mechanical strength and pathogenic impurities that can result in immunogenicity. However, synthetic polymers like poly lactic acid, polyvinyl alcohol, polyglycolic acid, etc., and their copolymers, on the other hand, are utilized widely in scaffold development due to their tunable mechanical properties and degradation rate control. Inorganic materials include tricalcium phosphate, metals, HAp, and their combinations replicating the bone mineral phase.

However, the development of hydrogels and scaffolds with better bioactivity, osteoconductivity and osseointegration capabilities in combination with high toughness value is very difficult to achieve. A team of researchers developed a robust and mechanically tougher osteoconductive hydrogel in a recent study. They followed a single-step micellar copolymerization of acrylamide and urethacrylate dextran after which they performed the *in-situ* mineralization of Hap nanocrystals. They demonstrated that the mineralized HAp concurrently enhances the mechanical and osteoconductivity properties. In comparison to pure PAAm hydrogels, the resulting HAp mineralized PAAm/Dex-U hydrogel (HAp-PADH) possessed improved fracture resistance of around 2300 Jm^2^ and an unusually high compressive strength of 6.5 MPa. They showed the enhanced osteogenic differentiation potential of the mineralized HAp layer that improved the osteoblast cell adhesion and proliferation *in-vitro*. They have concluded that HAp combination with PADH possesses an excellent potential for bone tissue repair and regeneration *in-vivo* when tested in a rabbit model of femoral condyle defect.

Similarly in another study, the synergistic effects of bioactive glass and halloysite nanotubes (i.e., BG/HNT) were assessed over the physicochemical and bioactive properties of polyacrylamide/poly (vinyl alcohol) (PMPV) based nanocomposite hydrogels. They applied the *in-situ* free-radical polymerization and a freeze-thaw method to successfully create a double-network hydrogel made up of organic-inorganic components. Bioactivity was tested for the developed scaffolds after immersion in the simulated bodily fluid (Ca/P: 1.21 0.14) and noticeably improved dynamic mechanical characteristics with compressive strength of 102.1 kPa at 45% of strain and stiffness of 3115.0 N/m at 15% of strain were revealed with enhanced biomineralization capacity of PMPV/BG/HNT. They demonstrated hFOB1.19 osteoblast cells growth and attachment over the developed hydrogels and concluded their efficacy for low-load bearing bone tissue.

Several researchers have applied additive manufacturing, bone-healing materials and functionally graded structures for bone regeneration and treatment of bone tumours. In a recent study, a team has developed novel AM-prepared polyvinyl alcohol/sodium alginate/hydroxyapatite hydrogel composite scaffolds at low temperatures. For which they placed different concentrations of magnetic graphene oxide/Fe_3_O_4_ nanoparticles onto the scaffolds. Characterization of the developed scaffolds showed their improved moldability and stronger hydrogen bonds between the composite material. They demonstrated that by adjusting the MGO’s composition and the strength of an external alternating magnetic field, thermal effects can be controlled. They tested *in-vitro* bone mesenchymal stem cell activity over these scaffolds which showed improved cell growth while the *in-vivo* study was performed which showed that the scaffolds exhibited favourable anti-tumour properties and efficient magnetothermal conversion *in-vivo*.

Bioceramics also play an essential role in improving the poor osteogenic characteristics and mechanical properties of the scaffolds for bone tissue repair and regeneration. Recent study conducted by a team demonstrated the application of free radical polymerization, to develop CaO.40SiO_2_.(12-y)P_2_O_5_.ySeO_3_.5MgO hydrogels containing polyacrylamide-carboxymethylcellulose. They enhanced the poor osteogenic property, controlled the rate of breakdown, and improved the mechanical stability by adding strontium and selenium doped xSrO. They achieved considerable apatite growth on the first day, and samples doped with bioceramics displayed outstanding bioactive behaviour, which was considered a factor for bone tissue regeneration and bone repair from flaws. Hydrogels impregnated with strontium and selenium and doped bioceramics showed an inhibitory effect on the human osteosarcoma MG63 cell line. Selenium and strontium-doped bioceramics offered a favourable environment for the cellular proliferation, adhesion, and excellent alkaline phosphatase activity of the MC3T3-E1 osteoblast cell line. Synthesized hydrogels represented unique osteogenic capabilities and therefore they may be used for bone cancer patients’ recovery as well as for the regeneration of hard tissues.

This article discusses in detail the fabrication of composite matrix and the potential ways for enhancing the bioactivity of scaffolds by doping and its advantages.

## 2 Bone tissue: ECM and its healing mechanism

Bone regeneration is the process of replacing bone tissue that has been damaged or lost due to trauma, injuries, cancer, or congenital defects. The extracellular space of the bone is filled with the ECM, a non-cellular, 3D material that cells release. Particular proteins and carbohydrates make up its structure. It is a complex, dynamic bio-environment with carefully controlled mechanical and biochemical properties. ECMs play a role in controlling cell attachment, proliferation, and responses to growth hormones in bone. They also significantly affect differentiation and, subsequently, the structural and functional attributes of the mature bone. Osteoblast-lineage cells, including MSCs, osteoblasts, and osteocytes, as well as osteoclasts, can both produce new bone and absorb existing bone when exposed to bone ECM. As bone regenerative medicine has advanced, researchers have become more interested in the osteoinductive and osteoconductive potential of ECM-based polymeric scaffolds.

Each type of tissue’s ECM develops with distinct composition and architecture (Frantz et al., 2010). The ECM provides consistency and flexibility to the body tissues and organs in terms of controlling their growth, activity, and homeostasis. It is constantly restructured as the wide range of receptors, growth regulators, and the pH of the native surrounding changes (Bonnans et al., 2014; Mouw et al., 2014). The fourth component in the evolution of BTE is thought to be the ECM (Ravindran et al., 2012). 60% of the bone matrix is made up of inorganic, and 40% of it is organic substances.

### 2.1 Major components of bone ECM

#### 2.1.1 Organic ECM

Collagenous proteins are found in collagen. The organic ECM in bones is mostly composed of collagen types I, III, and V. The principal function of collagens is to supply mechanical stability and support but to also act as a framework for osteocytes (Saito and Marumo, 2015). 90% of the collagen in bone tissue is type I collagen, which assembles into triple helices of polypeptides to create collagen fibrils. In order to create higher-order fibril bundles and fibers, these fibrils engage in interactions with other collagenous and non-collagenous proteins (Varma et al., 2016). Less common collagen types III and V control the size of type I collagen fibers and the process of fibrillogenesis (Garnero, 2015). The mechanical characteristics of collagen keep the polypeptide chains in a neatly structured fibril framework, depending on the inter and intra-chain crosslinks. Bone strength is significantly influenced by collagen. The ECM is altered by type I collagen deficiency or collagen structural mutations, which significantly raises the risk of fracture (Fonseca et al., 2014).

#### 2.1.2 Non-collagenous protein: Proteoglycans

Proteoglycans are defined as glycosaminoglycan (GAG) residues that have been covalently attached to the core of the protein molecule. Keratan sulfate, chondroitin sulfate, heparan sulfate, and dermatan sulfate are among the six varieties of GAG residues discovered in proteoglycans (Walimbe and Panitch, 2020). The bone has a significant family of small leucine-rich proteoglycans including biglycan, decorin, keratocan, and asporin. SLRPs are extracellular proteins secreted by cells that collaborate with cell-surface receptors and cytokines to regulate both healthy and unhealthy cellular functions. SLRPs are involved in all phases of bone development, such as cellular multiplication, osteogenesis, mineral deposition, and bone remodelling (Kirby and Young, 2018). SLRPs also control the collagen fibrillogenesis process. Dysregulation results in flaws in the structure and production of collagen and leads to fibrosis brought on by either orthopaedic traumas or genetic deficiencies (Moorehead et al., 2019). They control the collagen fibrillogenesis process, whose dysregulation results in flaws in the structure and production of collagen and leads to fibrosis brought on by either orthopaedic traumas or genetic deficiencies (Moorehead et al., 2019). The class I SLRPs biglycan and decorin each comprise dermatan or chondroitin sulfate GAG chains. Biglycan is expressed throughout the growth and mineralization of cells, whereas decorin is continually expressed once the bone matrix has begun to form. Keratocan plays a role in controlling the pace of mineral deposition and bone formation. It is mostly expressed in osteoblasts (Coulson-Thomas et al., 2015). It has been demonstrated that type I collagen and asporin, another SLRP component, bind together to induce collagen mineralization (Kalamajski et al., 2009). As a result, SLRPs are crucial for preserving bone homeostasis.

#### 2.1.3 Inorganic ECM

HA is a substance that promotes the production of bone tissue and is simple to combine with polymeric materials (Amini et al., 2012). Since the 1950s, HAp has been employed as an unreactive scaffold for filling and processing bone abnormalities in regenerative scientific knowledge (Dubok, 2000). Due to its strong osteoconductivity and biocompatibility, the calcium phosphate bio-ceramic [Ca_10_(PO_4_)_6_(OH)_2_] has been ubiquitously used as scaffolds in BTE. It is a well-known bioceramic that is present in significant amounts in bone and teeth (Amornkitbamrung et al., 2020). HAp serves as one of the most extensively utilized bio-ceramics in BTE because it exhibits physiochemical characteristics that are remarkably similar to carbonate apatite, the major inorganic constituent of bone tissue (Yuan et al., 1999; Ripamonti et al., 2009a; Pepla et al., 2014). Due to its advantageous biological characteristics, such as biological compatibility, bio-affinity, bio-activity, and osteoconduction (Habibovic et al., 2008), HAp bioceramics are frequently employed as artificial bone substitutes.

## 3 Healing of fracture: Mechanism

A fracture is a break in the internal structure of the bone cortex that causes damage to soft tissue. After the fracture, secondary healing starts, and it entails four stages: the development of a hematoma, a fibrocartilaginous callus, a bony callus, and bone remodelling. Up to 10% of all fractures may experience failed or delayed healing, which may be brought on by a variety of conditions, including comminution, infection, malignancy, and interrupted vascular supply. All these processes are discussed below in this article.

### 3.1 Phases of healing

#### 3.1.1 Hematoma formation

This phase starts right after the fracture from day 1 to day 5. A hematoma forms surrounding the fracture site as a result of the blood arteries supplying the bone and the periosteum being torn during the fracture. The hematoma clots, creating a framework that is eventually used during healing. Pro-inflammatory cytokines such as tumor necrosis factor-alpha, bone morphogenetic proteins, along with interleukins are released as a result of the bone injury (IL-1, IL-6, IL-11, IL-23). These cytokines draw macrophages, monocytes, and lymphocytes by stimulating the vital cellular biology at the location. These cells collaborate in order to remove necrotic, injured tissue and to aid the healing process by releasing cytokines such as vascular endothelial growth factor.

#### 3.1.2 Fibro-cartilaginous callus formation

When VEGF is released, angiogenesis occurs and fibrin-rich granulation tissue begins to form within the hematoma. More mesenchymal stem cells are brought in, where they start to develop into fibroblasts, chondroblasts, and osteoblasts under the control of BMPs between day 5 and day 11. Chondrogenesis begins, resulting in the formation of a collagen-rich fibro-cartilaginous network spanning the fatigue crack ends and a sleeve of hyaline cartilage all around it. The osteoprogenitor cells also lay down a layer of woven bone next to the periosteal layers at the same time.

#### 3.1.3 Bony callus formation

Endochondral ossification starts to take place in the cartilaginous callus between day 11 to day 28. When RANK-L is expressed, it encourages chondroblasts, chondroclasts, osteoblasts, and osteoclasts to differentiate further. As a result, the cartilaginous callus is resorbed and begins to calcify and the patterned woven bone is still being deposited subperiosteally. Mesenchymal stem cells can continue to migrate as the newly created blood arteries continue to multiply. An immature bone callus that is firm and calcified forms at the conclusion of this stage.

#### 3.1.4 Bone remodelling

The phrase “coupled remodelling” refers to the recurring remodelling of the hard callus that occurs as a result of the osteoblasts’ and osteoclasts’ ongoing migration after day 18 and it lasts for months. Osteoclast-driven bone resorption and osteoblast-driven bone formation coexist in this “coupled remodelling” process. Compact bone eventually replaces the callus’s centre, while lamellar bone eventually replaces the callus’ margins. Alongside these changes, the vasculature undergoes significant remodelling. The typical bone structure eventually regenerates after a protracted, months-long process of bone remodelling (Frost, 1989; Ripamonti et al., 2009b; Parvizi, 2010; Marsell and Einhorn, 2011; Kostenuik and Mirza, 2017).

**FIGURE 1:**
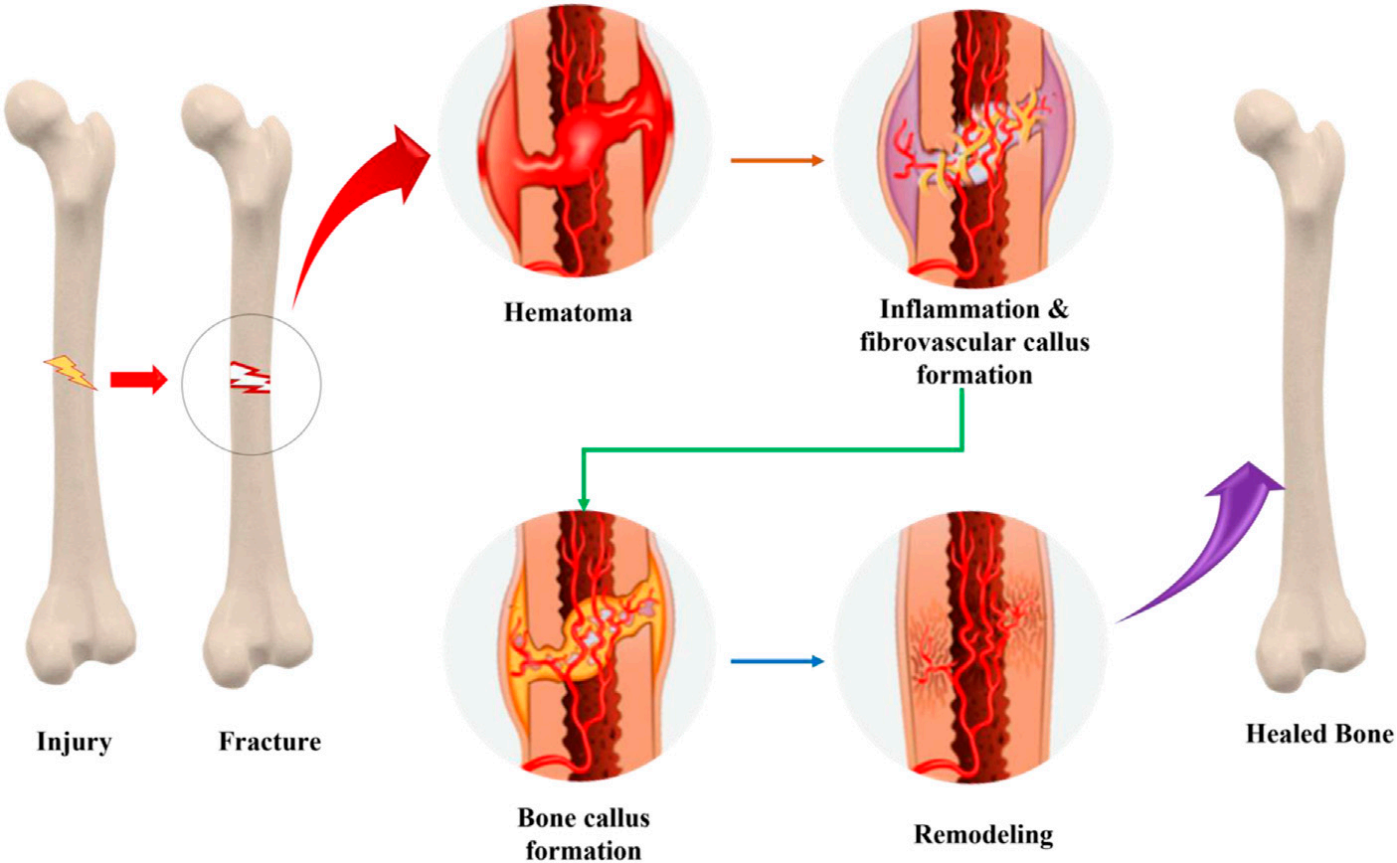
Process of fracture healing and mechanism involved in complete bone healing.

**FIGURE 2:**
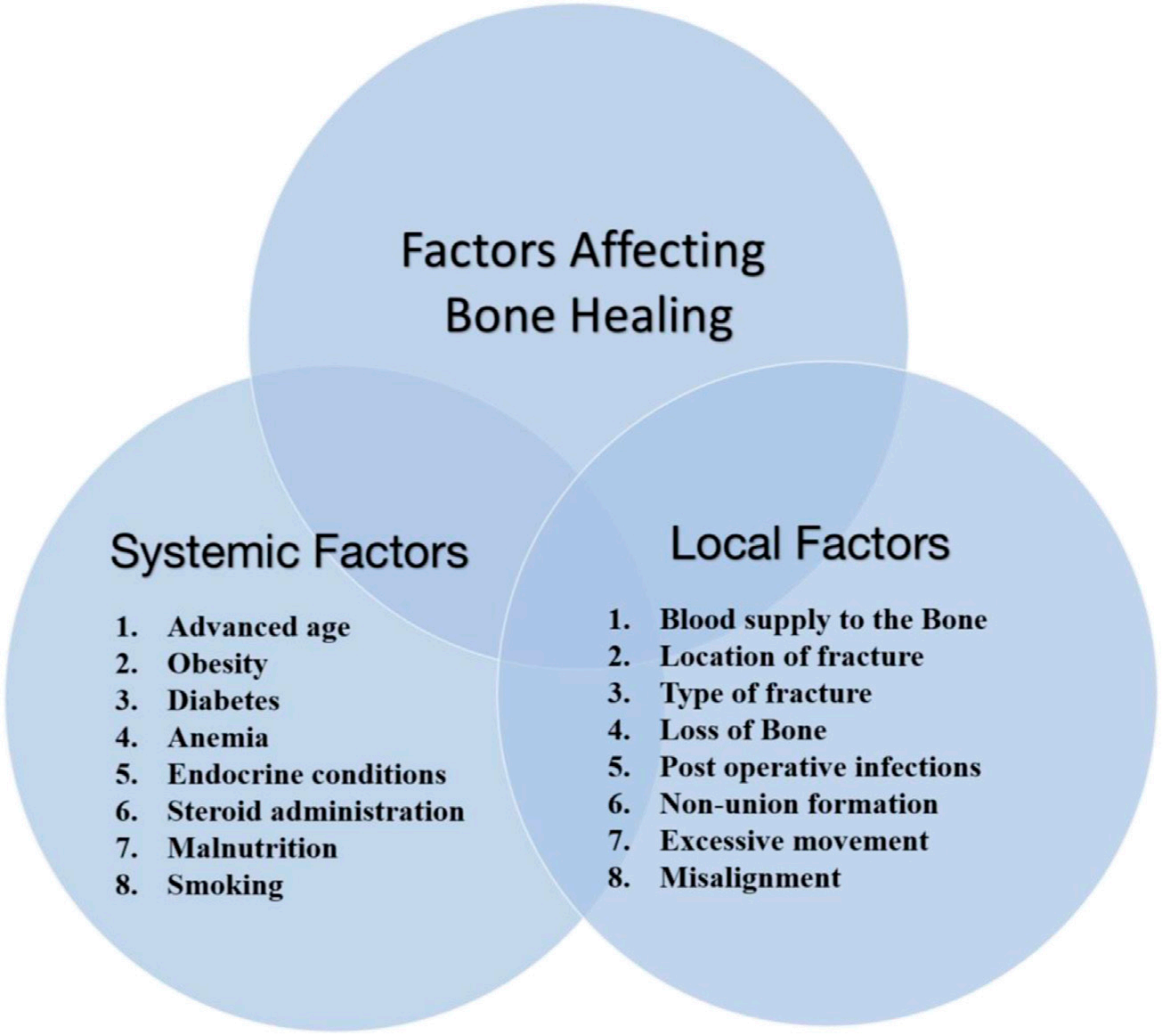
Factors responsible for fracture healing, including local and systemic factors.

Endochondral ossification is used to describe the transformation of cartilage to bone. As previously stated, this occurs when a bony callus forms, replacing the recently formed, collagen-rich cartilaginous callus with juvenile bone. This process, which involves the bony skeleton substituting the hyaline cartilage prototype, is also required for the foetus to develop long bones. The second type of ossification, intra-membranous ossification, also occurs in the developing foetus. During this process, mesenchymal tissue (primary connective tissue) is transformed straight into bone without cartilage as an intermediary. The flat bones of the cranium are where this process occurs (Berendsen and Olsen, 2015). The whole process from fracture to the complete healing of bone is explained in Figure 1. Figure 2 explains the factors involved in fracture healing including both local and systemic factors.

Given the high mortality and morbidity associated with fractures, an interdisciplinary approach is crucial for successful outcomes (Einhorn, 2005; Bishop et al., 2012; Karpouzos et al., 2017). The interprofessional team can employ various strategies to encourage/promote fracture restoration, such as calcium salts, protein molecules, and vitamins C and D as examples of dietary supplements. Ultrasound, electromagnetic, and electrical bone stimulators are all possible. Further study is needed in this area because it is still unclear whether these strategies are currently effective. Bone is used in a bone graft procedure to act as a framework of the scaffold for the developing bone. The patient’s body (an autograft) or a deceased donor’s body provides this graft (allograft) (Zhang and Williams, 2019). But there are still chances of immune rejection, which may lead to a necessary surgical procedure. For such conditions, ceramic and polymeric composite materials can be a good choice. They are biocompatible, mechanically stable, and also bioresorbable; therefore, they will support bone regeneration. This article discusses natural and synthetic polymeric composite matrices in detail, their advantages, and associated challenges.

## 4 Natural polymeric composite matrix: Advantages and challenges

TE is a multi-disciplinary field that develops bio-mimetic equivalents that reinstate, maintain, or enhance tissue functioning using precepts and developments from the biomedical sciences and technology (Montoya et al., 2021a). Biomaterials for TE applications can be defined as “a material designed to take a form that can direct, through interactions with living systems, the course of any therapeutic or diagnostic procedure” (Zhang and Kohn, 2012). Polymeric materials are nevertheless the most prevalently used biomaterial category in the fabrication and design of scaffolds for biomedical applications. They are excellent contenders for the construction of artificial bone and other body tissue scaffolds owing to their structural and bio-mechanical properties, appropriate biodegradation rates, and biocompatibility that perfectly approximates those of protein molecules in both soft as well as hard tissues (Mao et al., 2021). Scaffolding frameworks for BTE are porous materials employed to promote cellular attachment, growth, and multiplication while supporting the regeneration of new bone tissue (Lei et al., 2019). The selection of biomaterials or their hybrids used as scaffolds is critical for BTE applications. The usage of different materials and their composites in biomedical treatments has recently grown rapidly. The therapies for various bone-related ailments and disorders using biodegradable materials composites is one domain currently expecting to receive substantial research attention from the scientific community.

Biopolymers, the most widely accepted component of TE used to restore or replace or regenerate injured human body parts, are divided into natural or synthetic polymers (Taghipour et al., 2020). Polymers synthesized by biological systems, including microbial cells, plants, and animals, are classified as natural polymeric materials. Natural polymers have numerous applications, including adhesive bandages, absorbent materials, primed cosmetic products, therapeutic drug delivery, and clinical scaffolds (Titorencu et al., 2017). Natural or biological polymers can further be classified as protein- or polysaccharide-based polymers. The most intensively investigated naturally-derived polymeric materials for BTE are collagen, gelatin, chitosan, hyaluronic acid, silk fibroin, and many more (Kim et al., 2017). These natural polymers have their own sorts of advantages and disadvantages during the bone regeneration process and are briefly discussed in Table 1. These biopolymers have been blended in a plethora of forms, such as 3D porous scaffolds, hydrogels, nanofibrous scaffolds, films, microspheres, sponges, and composites (Han et al., 2017; Kim et al., 2017; Ikono et al., 2019; Filippi et al., 2020; Yao et al., 2020).

**TABLE 1:** Advantages and challenges of natural polymeric-based composite matrix for BTE.

Polymer	Composite matrix	Advantages	Challenges	References
1. Collagen	Col (Type I) + Silica bio-compositesChitin + hydroxyapatite + collagen scaffolds (CHCS)	Excellent biocompatibility and biodegradable; natural component of ECM; non-toxic; bio-adhesive; highly hydrophilic; mimic bone ECM topography; bio-functional; hemostatic; low immunogenicity; good permeability.	Limited osteoinductive ability; poor mechanical strength; poor structural stability; high biodegradability; costly.	Zhang et al. (2018a), Lin et al. (2019a), Lin et al. (2019b), Xing et al. (2021), Alvarez Echazú et al. (2022)
2. Gelatin	Gel + PCL + nHApGel + Bioactive glass scaffolds	High biocompatibility and biodegradability; high water solubility; low antigenicity; possesses anti-thrombogenic properties; the presence of RGD sequences allows better cell adherence.	Poor mechanical properties; less stable; highly biodegradable; brittleness; lack thermal stability	Raucci et al. (2019), Thomas and Bera (2019), Gautam et al. (2021)
3. Silk Fibroin	Silk Fibroin + carboxymethylcellulose chitosan cellulose nanocrystals + strontium substituted hydroxyapatite	Biocompatible; slower degradation; biodegradable; excellent mechanical stability; water-based processing; good toughness and ductility.	Limited availability; highly brittle; the presence of residue contaminants.	Ma et al. (2018), Zhang et al. (2019a), Chen et al. (2022)
	SF + Octacalcium phosphate + sodium alginate			
4. Chitosan	Ch + SF + HAp + β-tricalcium phosphateCh + Chondritin sulphate + nano-bioglass	Biocompatible; good biodegradability; possesses anti-microbial properties; non-toxic; cationic nature; owns the hemostatic property; less costly; abundantly available	Water insolubility; immunogenic nature; slow osteoconductive property; low mechanical strength and stability	LogithKumar et al. (2016), Singh et al. (2020a), Lu et al. (2022), Ribeiro et al. (2022)
5. Chondroitin sulfate	Ch + Strontium CS (Ch-SrCS)CS + Ch + NBG	Biocompatible; non-toxic by-products; non-immunogenic; possess anti-inflammatory & anti-oxidant properties; better bone regenerating properties; easily available	Water insolubility	(Kwon and Han, 2016; Sharma et al., 20221193; Xu et al., 2021; Singh et al., 2019)
6. Alginate	Alg + polyvinyl alcohol + Magnesium diboride (MgB_2_) Alg + (HAp) aerogels	Highly biocompatible; biodegradable; easy to functionalize; simple gelation methods; chelating ability; excellent encapsulation capacity; easy to mold in different forms, including fibers, sponges, etc.	Poor mechanical properties; leaching of encapsulated drugs; challenging to handle and sterilize.	Abhinandan et al. (2021), Iglesias-Mejuto and García-González (2021), Sahoo and Biswal (2021)
7. Cellulose	Regenerated cellulose (rCL) + Ch Carboxy methyl cellulose + Alg + *Spinacia oleracea* (SO) + *Cissus quadrangularis* (CQ) extract (CMC/Alg/SO/CQ)	Biocompatible; non-toxicity; easily available; inexpensive; bio-degradability.	Longer renewal time; poor osteointegration; low biodegradability in the human body.	Hickey and Pelling (2019), Sharmila et al. (2020), Maharjan et al. (2021), Janmohammadi et al. (2023)
8. Hyaluronic acid	HA + Gel + elastin + BMP-2-conjugated carbon dots (BMP2-CDs) Lysine modified HA + Col + Ch	Highly biocompatible; biodegradable; high water solubility; natural component of ECM; excellent viscoelasticity; promotes cellular activities and growth; easy to functionalize	Poor mechanical strength; difficult to form fibers; costly	(Li et al., 20192019; Gilarska et al., 2020; Xing et al., 2020; Moztarzadeh et al., 2021)
9. Fibrin	Fibrin + graphene oxide (GO) loaded hydrogels Fibrin + Alginate + Calcium phosphate (CaP) porous scaffolds	Biocompatible; biodegradable; improved cell-matrix interaction;	Poor mechanical properties; instability; rapid degradation	Kohli et al. (2021), Pathmanapan et al. (2020), al Kayal et al. (2020)
10. Starch	Starch + nano-GO nanofibrous scaffoldsStarch + PCL + silicon-loaded wet-spun fibrous scaffolds	Excellent biocompatibility; biodegradable; non-toxic; good mechanical properties; low cost; readily available	Low surface area; difficult processing; brittleness	Rodrigues et al. (2012), Miculescu et al. (2017), Wu et al. (2017)
11. Gellan gum (GG)	3D-printed gelatin methacrylate (GelMA) + gellan gum methacrylate (GGMA) GG + *Gallus gallus* demineralized bone powder (GDBP)GG + HAp bilayered hydrogel	Biocompatible; non-toxic; posses flexibility and elasticity; highly stable; biodegradable	Insufficient mechanical properties; lack of cell adhesion sites; high gelation temperature	Pereira et al. (2018), Kim et al. (2019a), Li et al. (2022a)
12. Dextran	Dextran + Bioglass ceramic nanoparticles (nBGC) loaded hydrogel(Dex-U) + Polyacrilamide + nHAP loaded hydrogel	Biocompatible; biodegradable; hydrophilic polymer	Poor mechanical properties; costly	Ghassemi et al. (2018), Nikpour et al. (2018), Fang et al. (2019)

Protein-based polymers, unlike polysaccharides, contain amino acid (AA) sequences that are traditionally coupled with cell adhesion *via* integrin-binding domains or RGD sequences (Guo et al., 2021). As a result, cell attachment and osteoconductivity in polysaccharide-based polymeric scaffolds must be improved through surface chemical functionalization, blending with osteoconductive components, and integration of cell adhesion protein sequences or by blending them with protein-based polymers (Yang et al., 2021). Bio-composites are created by blending two or more biomaterials to improve cytocompatibility and mechanical characteristics of scaffolds for various applications (Goonoo et al., 2013; Basha et al., 2015; Mao et al., 2018). Various studies have been conducted over the last few decades by blending various polymers and other ceramic-based components to achieve the desired cell behaviours and mechanical strength to promote bone tissue renewal and repair, as summarized in Table 1.

## 5 Synthetic polymer composite matrix: Advantages and challenges

In general, polymeric materials and their composites provide greater control over scaffolds’ physical and chemical properties, including pore size and shape, porosity, hydrophilicity, cytocompatibility, non-toxicity, enzymatic activities, and inflammatory response (Liu and Ma, 2004; Prasad, 2021). The physicochemical properties of scaffolds and the biomaterials used in their construction significantly impact their performance in transplantation, and as a result, various biomaterials are being employed in BTE. The physicochemical and biological attributes are among the factors to consider when selecting biomaterials for therapeutic bone implants. Ceramics, polymeric materials, metals, and various nano-composites are frequently reported classes of materials for hard tissue regeneration (Koons et al., 2020; Ye et al., 2020).

Synthetic polymers were developed and are extensively used in the designing and construction of scaffolds for bone tissue repair and regeneration due to their superior mechanical characteristics. Multiple synthetic materials, such as PLA, PLGA, PVA, and others, have been extensively used for bone tissue regeneration and remodelling (Donnaloja et al., 2020). Also, each type of above discussed synthetic material has its own set of benefits and drawbacks, and high-performance composite polymeric materials are constantly being researched and are discussed in Table 2. They include more possibilities for customizing and modifying their physiochemical behaviour, mechanical stabilities, cell binding domains, and immobilization of different NPs, drugs, or bioactive compounds (Amani et al., 2019). Introducing RGD peptide sequences or growth factors to polymer matrix could further significantly change their properties for cell attachment and proliferation (Zhang et al., 2011; Udomluck et al., 2020).

**TABLE 2:** Advantages and challenges of synthetic polymeric-based composite matrix for BTE.

Polymer	Composite matrix	Advantages	Challenges	References
1. Polylactic acid	PLA-loaded with HAp + PCLPLA + PCL + Tetracycline hydrochloride (TCH)	Biocompatible; biodegradable; non-toxic; non-inflammatory; stimulates cellular activities; FDA approved	Biological inertness; slow degradation rate; hydrophobic property	Narayanan et al. (2016), Farzamfar et al. (2019), Kareem et al. (2019), Idumah et al. (2021)
2. Polyglycolic acid	PGA + SFPGA + HAp	Biocompatible, faster biodegradation rates; non-toxic by-products; stable thermal and mechanical properties; supports cell adherence; FDA approved	Induce inflammatory response; high cost	Kim et al. (2019b), Budak et al. (2020), Yeo et al. (2021)
3. Poly (lactic-co-glycolic acid)	PLGA + nHApPLGA + HAp microspheres	Biodegradable; promotes cell adhesion; FDA approved; mechanical properties can be adjusted	Exhibit immunogenicity; the presence of contaminants	Kong et al. (2017), Martins et al. (2018), Babilotte et al. (2021), Wei et al. (2022)
4. Polyethylene glycol	Gelatin methacryloyl (GelMA) + poly(ethylene glycol) diacrylate (PEGDA) + nHAp PEG + PCL + roxithromycin (Rox)	Biocompatible; low immunogenicity; high water solubility; non-toxic; easy to mold in different forms; FDA approved	High degradation rates; lack of cell adhesion ability	Kong et al. (2017), Bai et al. (2020), Sreekumaran et al. (2021)
5. Polyvinyl alcohol	Ch + PVA + NBG + nano-zinc oxide (CPBZ) Gel + PVA	Excellent biocompatibility,non-toxic, chemical and thermal stability, good mechanical stability and flexibility, high water solubility; possess adhesive properties and cost-effective	Inadequate elasticity, poor hydrophilicity, poor cell adhesion properties	Kumar and Han (2017), Baker et al. (2012), Mallakpour et al. (2022), Christy et al. (2022), Thangprasert et al. (2019)
6. Poly ɛ-caprolactone	PCL + bioactive glass nano-particles (BGNPs) + simvastatin (SIM) PCL + CaCO_3_ + ALP	Biodegradable, non-toxic by-products, FDA approved	Hydrophobic; limits cell attachment	Surmenev et al. (2019), Dwivedi et al. (2020), Ghorbani et al. (2021), Saveleva et al. (2021), de Souza and Moraes (2022)
7. Poly(propylene fumarate)	PPF + Zn-doped HAp scaffolds 3D-printed PPF + HAp scaffolds	Biocompatible; biodegradable; non-toxic byproducts; good mechanical properties	Viscous liquid at RT; difficult processing	Wang et al. (2006), Trachtenberg et al. (2017), Cai et al. (2019), Li et al. (2022b)
8. Polyurethane	PU + HAp + β-cyclodextrin loaded porous scaffolds 3D-printed Layfomm (PU + PVA) scaffolds	Highly biocompatible; biodegradable; hemo-compatible; non-toxic; superior mechanical properties; easy to process	Poor thermal stability; toxic precursors used for PU synthesis	Ma (2008), Du et al. (2018), Cooke et al. (2020)

Furthermore, the polymers’ hydrophilic and hydrophobic properties could be customized to minimize immunogenicity and promote osteoconductivity. For instance, PLGA is lactic acid and glycolic acid copolymer (Castillo-Dalí et al., 2015). Their biodegradation rate is determined by the proportion of lactic acid to glycolic acid; consequently, it could be controllable and modified to meet their specific requirements. This may have an impact on their use in sustaining the delivery of therapeutic drugs and GFs at the site of orthopedic defects (Hines and Kaplan, 2013). Reportedly, NBG-coated PCL/PLGA composite scaffolds demonstrated improved compressive strength, *in-vitro* biocompatibility, sustained drug release, and increased MC3T3-E1 cell attachment and proliferation (Ensoylu et al., 2021).

## 6 Smart materials for bone tissue engineering

A biocompatible and biodegradable polymer-based scaffold provides superior mechanical and porous interconnected network, frame, and hierarchy structures with physiochemical properties for cell adhesion, proliferation, and cell-matrix interaction through the porous structures for tissue regeneration in TE. To date, most of the synthetic, natural materials and their composites have been explored for TE applications (Arif et al., 20221081; Biswal, 2021). Several methods and approaches for constructing bio-mimetic scaffolds with control over structure, shape, pore size, porosity, and architectural style for TE application domains have been devised (Turnbull et al., 2018; Kanwar and Vijayavenkataraman, 2021). To date, a wide variety of biomaterials have been employed in tissue engineering, a majority of which lack biocompatible properties and necessitate the cost-effective designing and construction of new smart materials for the highest performance. Smart materials are a material wherein one or more attributes can be changed simply by altering the surrounding environment (Zhang et al., 2018b; Jia et al., 2019). Additionally, smart biomaterials are distinct from conventional biopolymers in that they respond to both external and internal stimuli in their surroundings (Fu et al., 2021). These smart biomaterials can maximize therapeutic potential while minimizing unwanted complications by speedily recognizing and responding to the target tissue surroundings, impart therapeutic value whilst conserving physiologically normal cells and tissues, and thus improve the quality and performance of patient care (Zhang et al., 2018b; Zeng et al., 2019).

Furthermore, Montoya et al. (2021b) notably re-defined the concept of “smart materials” and postulated four levels of material smartness predicated on their inert, active, responsive, and autonomous behaviour. They also divide smart biomaterials into two types: those that respond to internal stimuli, such as surface topography, structural properties, and charge density, and those that respond to external stimuli, such as piezoelectricity, magnetic fields, ionic strength, and enzymes (Montoya et al., 2021b). The aforementioned external/internal stimuli produce heat or enhance osteoblasts to adhere, rapidly proliferate, and differentiate in polymeric scaffolds during bone regeneration and tissue repair. One well-known piezoelectric material with BTE applications is Polyvinylidene fluoride is used in restorative engineering because of its adaptability and cytocompatibility. Fernandes et al. (2019), for example, used a piezoelectric polymer, PVDF, and magnetostrictive CoFe_2_O_4_ NPs in a solvent cast method to create 3D magnetoactive porous structures. The use of magnetic stimuli increased the proliferation of preosteoblasts in this study. Bioelectrical signals and internal and external electrical stimulation play important functions in regulating cellular behaviour and bone restoration, and electrically conducting polymeric materials (such as polyaniline, CNTs, polypyrrole, etc.) have received a significant amount of attention as a good source of biomaterials (Guo and Ma, 2018). In the latest study, a construct made of functionalized Multiwalled Carbon Nanotubes, chitosan, and β-Glycerophosphate demonstrated improved electrical conductivity, mechanical stability, less cytotoxicity, and increased ALP activity (Gholizadeh et al., 2017). The preferential catalytic activity of particular enzymes activates enzyme-responsive materials (Ding et al., 2020). The key benefit of ERMs is that their stimulation does not necessitate environmental stimuli because the enzyme-mediated differences exist within the biological system, and enzymatic activities are governed by changes in the physiological environment, resulting in highly beneficial effects in enzymatic reactions (Ding et al., 2020). Zhang et al., for instance, developed a poly(L-lactic acid) nanofibrous construct embedded with a HP/miRNA polyplex enclosed in PLGA microspheres that demonstrated excellent cytocompatibility, non-toxicity, regulated two-stage release of miRNA-26a and *in-vivo* regrowth of the bone defects (Zhang et al., 2016a).

Moreover, highly developed processing techniques are essential for the production of SBMs. Biopolymers with the relatively low extent of smartness levels can enhance their interrelations with their surroundings and acquire some novelty by using innovative manufacturing methods such as 3D/4D bioprinting, electrospinning, blow spinning, and many other addictive manufacturing techniques. These techniques have attracted wide recognition owing to the potential of producing customized products with governed structure, distinguishable nano- or micro-morphology, and a higher level of alignment with defined physical attributes for BTE (Zhang et al., 2016b; Qasim et al., 2019a). Sharma et al. developed a 3D-printed PLA construct fortified with polydopamine-reduced graphene oxide for new bone tissue development (Sharma et al., 2022a). The study demonstrated the dependence of hMSCs cell growth on fiber direction and nano-coating. In one analysis, electrospun polyurethane urea nanofibrous scaffolds loaded with carboxyl functionalized CNTs (CCNTs)-doped nHAp illustrated superior mechanical properties, excellent cytocompatibility, enhanced expression of osteocalcin and ALP *in-vitro*, and impressive bone regeneration efficacy and non-toxicity *in-vivo* (Ghorai et al., 2022). Table 3 shows the recently developed smart materials like photosensitive, stimuli-responsive, electromechanical responsive and other advanced biomaterials with their applications for BTE along with their properties and fabrication technology.

**TABLE 3:** Smart materials and their applications in BTE.

Materials	Properties	Technique	Applications	References
1. Poly(lactide-co-glycolide) microspheres coupled with P-15	Highly porous structure, reduced degradation rate, enhanced cell growth, migration and proliferation rate due to coupling with P-15	Gas foaming	Bone Tissue engineering	Mittal et al. (2010)
2. Osteoimmunomodulatory biomaterial Heparin-modified gelatin nanofibers, and interleukin 4	Controlled release to modulate polarization of macrophages, reduced inflammation, and enhanced osteoblastic differentiation and bone regeneration	Self- assembled monolayer	DM-associated bone regeneration	Hu et al. (2018)
3. Shape memory smart scaffolds Poly(ε-caprolactone) and HAp nanoparticles loaded with BMP2	Predesigned, deformed for easier implantation into the defect site *via* minimally invasive surgery followed by expansion to adjust into an deformed bone defect	Sugar leaching	Bone regeneration	Liu et al. (2014)
4. (Photothermally controlled) smart scaffold Nano-hydroxyapatite/graphene oxide/chitosan scaffold	Killing human osteosarcoma cells under irradiation and enhanced osteogenesis in coordination with nHAp, good hemostatic effect and soft tissue restoration and repairing under irradiation	Lyophilization	Osteosarcoma treatment	Ma et al. (2020)
5. Piezoelectric Poly(vinylidene fluoride-trifluoroethylene)	Dynamic compression at 1 Hz frequency, improved MSC chondrogenesis	Electrospinning	Bone tissue engineering	Qasim et al. (2019b)
6. Piezoelectric HA/barium titanate	Periodic loading, enhanced osteoblast proliferation and growth due to electrical stimulation, very similar to the piezoelectric effects on human bone growth, modelling and reconstruction *in-vivo*		Bone tissue engineering	Tang et al. (2017)
7. N-isopropylacrylamide, pentaerythritol diacrylate monostearate, 2-hydroxyethyl acrylate, and vinyl phosphonic acid	Stimuli responsive, dually responsive macromers, Examine the effect of increasing vinyl phosphonic acid content	Free radical polymerization & thermo-gelation	Cellular delivery	Kretlow et al. (2010)
8. Dual-functionalized Mesoporous silica nanospheres	Drug release rate can be controlled continuously and remotely using single or dual stimuli, thin macromolecular coating acted as a rate modulator for regulating the diffusion kinetics of the drugs	Copolymerization	Thermo and electro-responsive drug delivery	Li et al. (2014)
9. PCL + BMP-2	Higher immobilization efficiency due to conjugation, controlled BMP-2 release, BMSCs upregulated growth and proliferation	Crosslinking and conjugation	Bone tissue regeneration	Zhang et al. (2010)

## 7 Biopolymeric modification for enhanced bone cell adhesion and growth

### 7.1 Modification strategies

**FIGURE 3:**
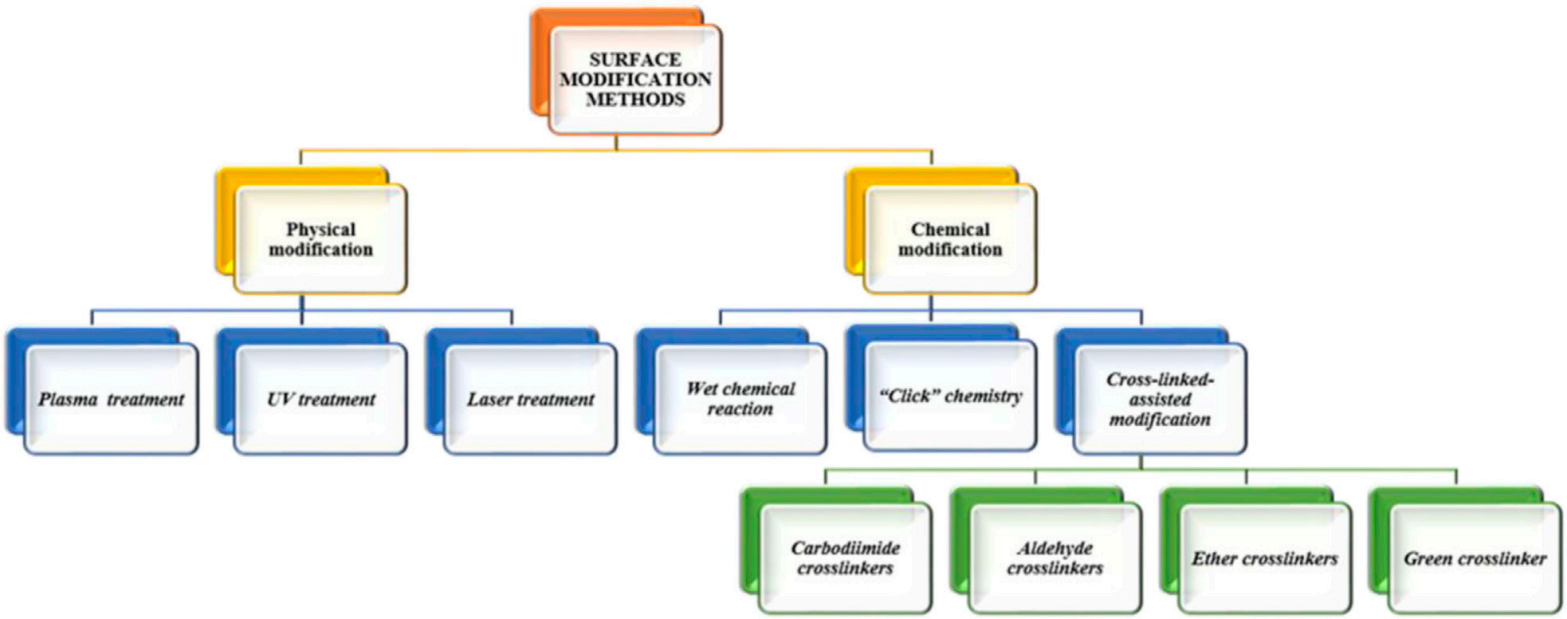
Schematic representation of several techniques for surface modification.

Biomaterials with novel biological functionalities are recognized as promising candidates for bone-regenerating materials. These materials aim to mimic the ECM at the nanoscale by imitating cues in topography, biological factors, and gene transport. Cell adhesion, cell proliferation, spreading, differentiation, and subsequent tissue development are all influenced by surface characteristics such as surface chemistry, net charge, roughness, hardness, and wettability. Thus, many recent studies have addressed surface modification of scaffolds to create physical and chemical properties for cell homing. The goal of biomaterial surface modification is to enhance selectivity. Coating or modifying the surface of biomaterials by a new functional group is one surface modification method. Alternative surface topologies are produced when functional groups are present, resulting in structures with minimum unfavourable interactions. Scaffold architecture is directly correlated to the binding capacity of functional groups, surface topology, as well as cell behaviours. The different types of surface modifications are broadly categorized into chemical modifications and physical modifications (Fabbri and Messori, 2017). Protein and cell adhesion are significantly influenced by the surfaces of polymeric scaffolds. To enable better protein and cell interactions, numerous strategies have been devised to give micrometre to nanometer size modifications in the surface architecture of scaffolds. One of the forthcoming techniques that offer increased biocompatibility while offering a protein delivery vehicle is chemical modification of polymeric scaffold surfaces (Katti et al., 2008). Chemical modification methods include wet chemical reaction (Duan and Wang, 2006), click chemistry (Bayraktar et al., 2012), crosslinkers assisted modification (Yang and Moloney, 2017). Further, the crosslinking-assisted modification encompasses of various crosslinkers like carbodiimide (Ikeda et al., 2010), aldehyde (Lin et al., 2016), ether (Miyagaki et al., 2019) and green crosslinking agents (Hubbe et al., 2015). In order to overcome the main drawbacks associated with chemical methods, which frequently require stringent process control, may result in environmental issues due to the chemical agents used, and may involve undesirable changes in the polymer surface morphology, surface treatment methods based on physical principles have been developed to introduce oxygen-containing functional groups on to polymer surfaces. These functional groups are primarily intended to improve adhesion, wettability, and printability. Today, the industrial level makes extensive use of some of the most popular physical surface modification techniques based on UV radiation (Sionkowska, 2014), plasma-induced treatments (Chu et al., 2002) and laser treatments (Jasso-Gastinel and Kenny, 2016). Different types of polymeric scaffold surface modifications are shown in Figure 3.

### 7.2 Factors influencing the surface properties

#### 7.2.1 Surface topography

The surface morphology or roughness of the substrate has been shown to influence cell adhesion. The fibres, pores, and pits that make up the surface of biological tissues are just a few examples of the broad range of morphological features found on their surfaces. This influence on cell activity is referred to as “contact guidance,” and a wide range of micro-morphological traits mediates it. Studies in this field have demonstrated that while micro-morphology mainly influences the shape of entire cells, nano-morphology primarily determines the sensing mechanism at the subcellular level. Surface roughness could be subdivided into many categories based on the irregular surface size: macro, micro, submicron, and nanoscale roughness. There is a wide range of cellular responses to surface roughness. Since there is plenty of room for cells to expand and proliferate within macroscopic irregularities, surface roughness has minimal impact on cell attachment behaviour. Surface roughness together at micron and submicron levels have opposing but positive effects on cell adhesion and proliferation. Zhao et al. (2014) discovered that the number of functional MG-63 cells on titanium discs with sub-micron surface roughness was lower than on flat nano-structured materials. Nano-roughness, on the contrary, has been shown to have a beneficial effect on cell adhesion, development, and maturation since it most closely mimics the morphology of real tissues. It has been illustrated that improving a biomaterial’s surface roughness at the nanoscale scale can improve cell adherence and proliferation on the rough surface, at least in human venous endothelial cells (Nguyen et al., 2016; Zhou et al., 2018) Pore size seems to be the key factor controlling cell adhesion. Previous research has demonstrated that nanoscale holes are highly susceptible to the formation of collagen fibers and ECM. *In-vivo*, larger pores have an impact on cell seeding, dispersion, adhesion, migration, and subsequent neovascularization. Large pore size would inhibit the adherence of cells. Researchers have found that the MG-63 cells adhere efficiently to the membrane’s surface, having a pore size range of 0.2–1 μm, while in the case of membranes with bigger micropores (3.0–8.0 μm), cells appear to be spherical in shape. Furthermore, cells differentiate more extensively when cultured on a membrane with pores between 5.0 and 8.0 nm, with the maximum differentiation occurring on the pore size of 8 nm (Schmitt et al., 2016; CM and O’Brien, 2010; Jeon et al., 2015).

#### 7.2.2 Physical properties

Bone cells, under native conditions, adhere to ECM fibers of varying hardness and flexibility. Bone ECM *in-vivo* has a rigidity of around 100 GPa. The elasticity and rigidity of fiber ECM are both controlled by the ratio of collagen to elastin. When cells apply stresses to the ECM and detect the ensuing gaps, they gain insight into the mechanical characteristics of the ECM. They can then adjust the local adhesion architecture, cytoskeleton components, and their overall state accordingly (Yang et al., 2017; Padhi and Nain, 2020). Even though cells appear to be more prone to adhere to hydrophilic substrate surface, wettability (hydrophobicity and hydrophilicity) can also influence surface protein adsorption and cell adhesion (Guo et al., 2016). Wei et al. (2022) showed that increasing the contact angle from 0° to 106° reduced the adhesion of osteoblasts. Fibroblast adhesion is significantly greater when the contact angle is between 60° and 80°. As a result, it demonstrates that the physical attributes of the material surface are critical in cell adhesion.

#### 7.2.3 Chemical Properties

**FIGURE 4:**
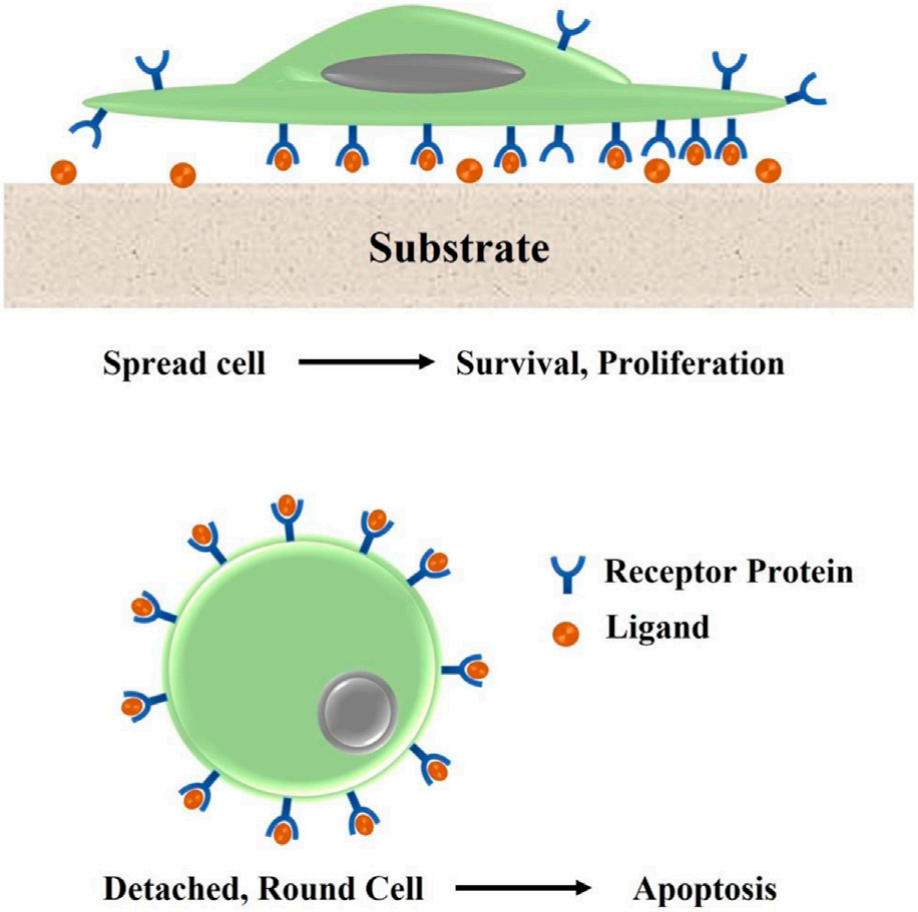
Immobilized ligand molecules that function as agonists of the ECM modify the surface. Non-immobilized ligands prevent cells from adhering to the scaffold surface, which causes apoptosis.

Multiple studies have established that surface chemistry plays a vital role in how cells adhere to different substrates. *In-vivo*, ECM provides cells with a wealth of chemical cues that direct their actions. Surface charges, surface energy, as well as bioactive substances are the most important chemical properties that influence cell adhesion (Kao et al., 2018). To quantify the unsaturated bond energy introduced by that of the surface material hanging bond, scientists have developed the concept of “surface energy”. Because of this, it has the potential to alter cellular function. For instance, the serum protein adsorption and cell adhesion that occurs upon contact between the polymer material surface and a biological fluid are both energy-dependent. The adherence and spreading of cells are promoted by surfaces having high free energy, whereas the opposite is true for surfaces with low free energy. Plasma treatment is also one of the techniques being widely used for altering the energy level of polymer surfaces (CG et al., 2006; Yongabi et al., 2020). Cell adhesion can also be affected by the polymers’ charge properties of adhesion surfaces. A substantial body of research shows that cells are more likely to cling to positively charged materials. Subtle alterations in cell activity can be induced by surface charge *via* the chemical functional groups of polymeric material (Terada et al., 2012). According to research published by Abarrategi et al. (2010), surfaces with varying charges (−CH_3_, −OH, −COOH, and −NH_2_ groups) controlled FN adsorption as well as integrin direct binding, along with OH > COOHNH_2_ > CH_3_ being the preferred pattern for MC3T3 osteoblast adherence to FN-coated surfaces. Figure 4 shows the mechanism of cell attachment to the biomaterial surface.

### 7.3 Techniques for surface modification for improved cell adhesion

**FIGURE 5:**
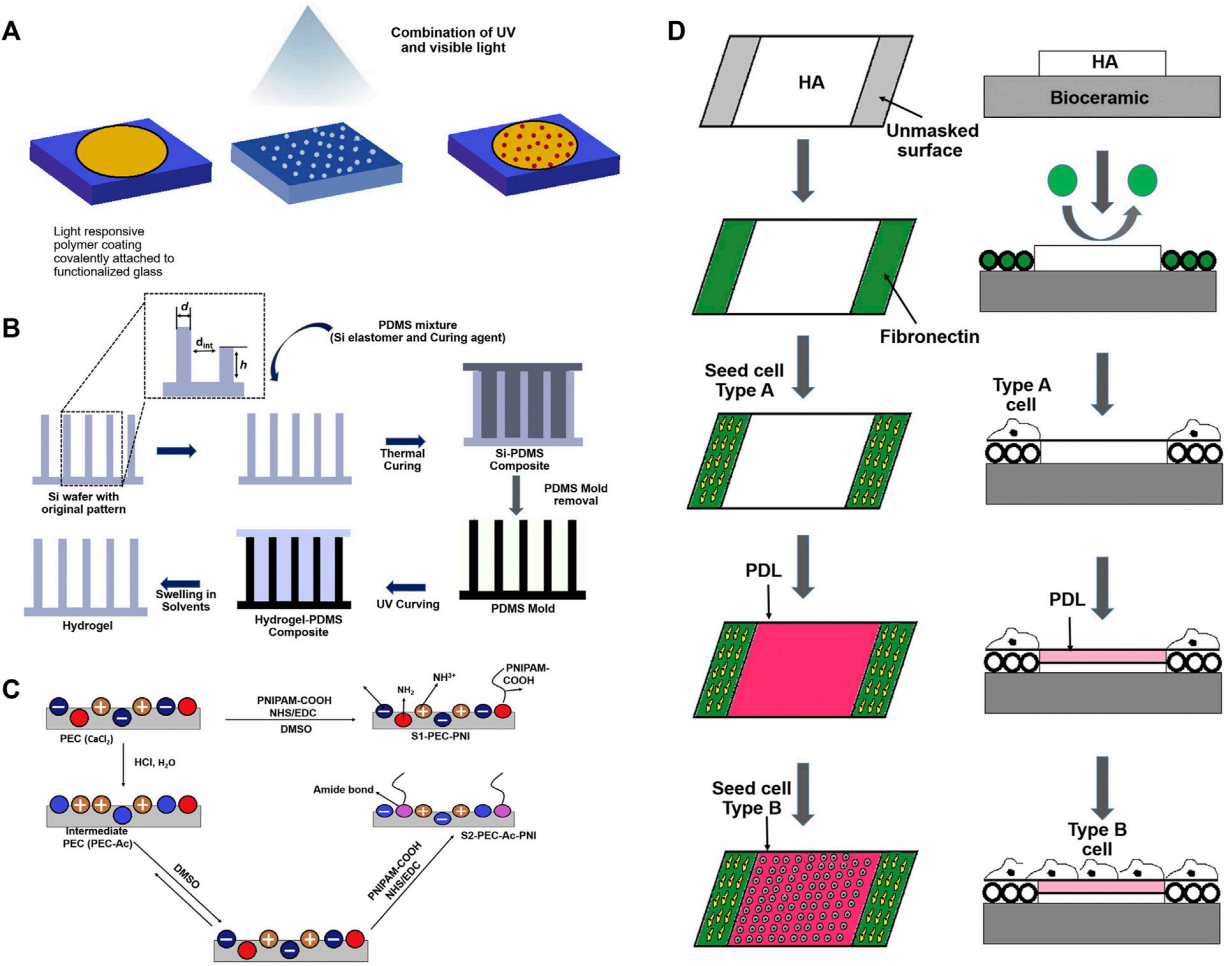
Different surface modification techniques of scaffolds for cell adhesion and proliferation: **(A)** Mask illumination; **(B)**; Soft lithography; **(C)** EDC-NHS Coupling; **(D)** Deposition of HA-PLL.

**FIGURE 6:**
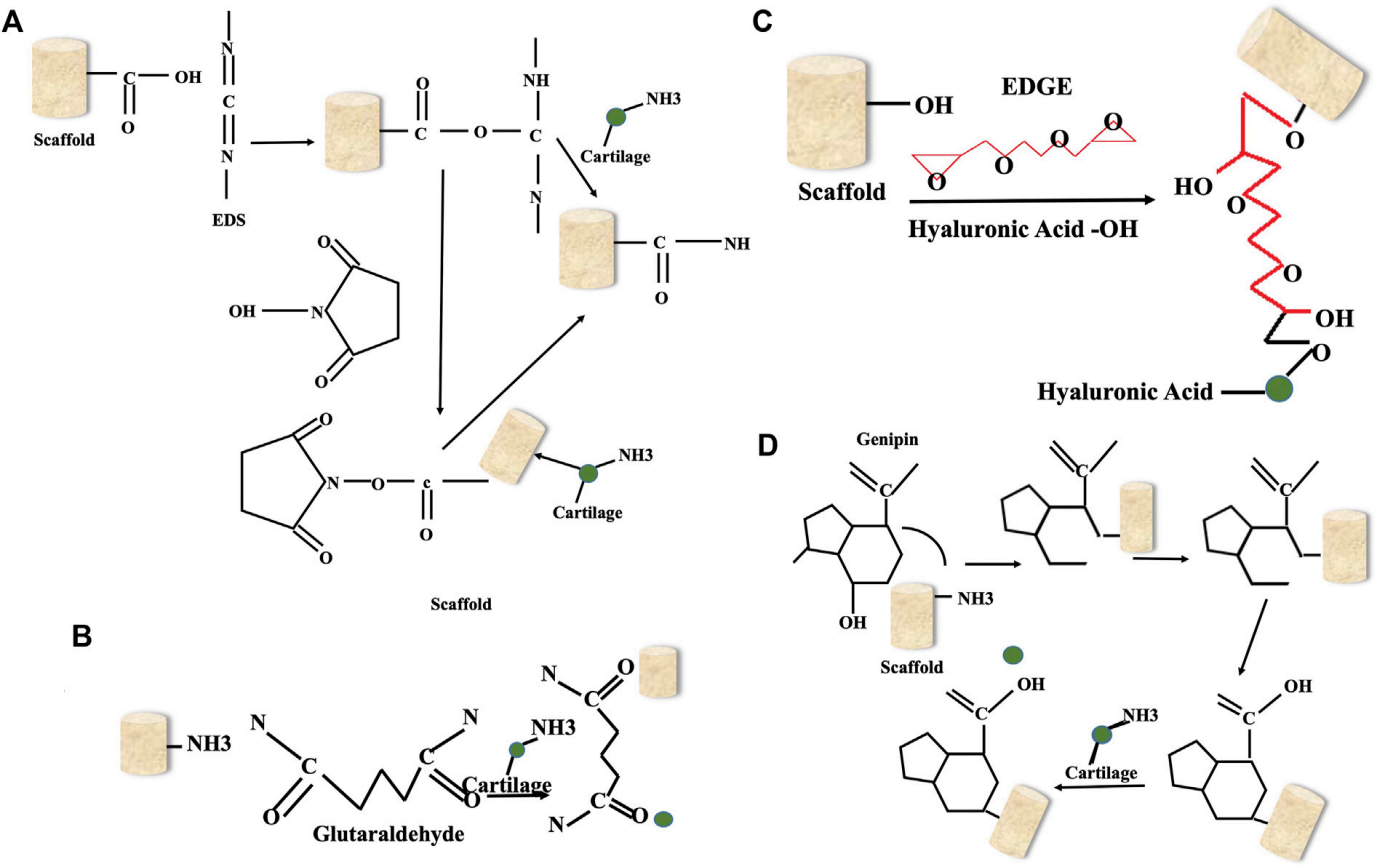
Different crosslinkers for bone tissue scaffolds: **(A)** EDC-NHS; **(B)**; Glutaraldehyde; **(C)** EDGE; **(D)** Genipin.

Several techniques for modifying cellular adhesion surfaces have been developed in an attempt to better comprehend the process by which cell-surface interacts, as illustrated in Table 1. While Figure 5 illustrates the surface modification methods involved in creating cell adhesion surfaces over the biomaterial surface. Figure 6 shows the various crosslinkers including natural and synthetic ones for bone tissue scaffolds. Table 4 shows various strategies for the development of cell adhesive surfaces.

**TABLE 4:** Different strategies for developing cell adhesion surfaces.

Techniques	Production methods	Advantages	Disadvantages
Self- assembled monolayers	Adsorption of an active chemical onto a solid substrate in a diluted solution results in the formation of ordered molecular structures	Greater hierarchy and orientation	Unique and especially treated solid surface is required
Polymer brush	The macromolecular structure is composed of polymer chains including one end securely inserted on a curved surface or plane	Significantly enhanced the substrate’s performance, displaying varied characteristics when ambient circumstances changed	Process complexity and the potential for material loss
Layer-by-layer assembly	Intermittent rinse steps after each successive deposition of interacting species on a substrate	Controlled layered structures, economical, fast, and easy methods	Rely upon centrifugation, require challenging scaling, and poor throughput assembling
Photolithography	Using a variety of energy resources to imprint patterns on a substrate surface, including electron beam, laser, as well as ultraviolet light	High accuracy	Sophisticated operation, expensive machinery
Electrospun fibers	Static electricity attracts the polymeric mixture or melts to the material membrane under high-voltage bias.	High level of orientation control precision and porous fiber structure	Issue of high pressure, susceptible to mechanical deformation
Spin coating	Deposition, rotation, rotation, and evaporation are the fundamental steps	Low pollution, high-performance costs, energy efficiency, and no coupling of process variables	Low rate of material usage, ongoing waste
3D bio-printing	The 3D layered polymeric structure is built from the ground up and printed with solvent biological materials, whereas the 2D patterned polymer layer is surface customized using computer-aided imaging methods.	High speed and accuracy	Poor cell survival rate, shear force

## 8 Cellular adhesion materials

Every material has its own property, and it causes different reactions in cells. The biocompatibility of materials is the most significant quality on the surface of cell culture. For example, Silicon is widely applied in bone tissue engineering due to its excellent cell adhesion and biological activity. But since silicon is also very unstable, metallic materials have been applied that offer higher strength, hardness, resistance to fatigue, and simplicity in processing and formation of stents in interventional treatments of bone. Likewise, Bioceramics are a class of materials offering low toxicity and biocompatibility. Therefore they are used in dental restoration, artificial hips, other bones, tooth roots, joints, bolts, etc. However, bioceramics possess biocompatible nature yet have a lower toughness value, leading to their application in combination with polymers with biodegradable and biocompatible nature for repairing and transplanting organs. These materials have exceptional and remarkable abilities in the field of tissue engineering; therefore, they are widely used by researchers and clinicians for bone tissue regenerative applications. Table 5 shows the different methods for modification of the scaffold’s surface.

**TABLE 5:** Recent research discusses various methods for scaffold surface modification.

Technique	Scaffold materials	Substrate	Cells used	Effect on cell adhesion and growth	References
LbL assembly	CH/HA	Titanium discs	MC3T3-E1	Coatings enhanced biomineralization, were biocompatible with pre-osteoblast cells, and had substantial anti-activity against *Streptococcus gordonii* infectious disease.	Govindharajulu et al. (2017)
	Collagen I/hyaluronic acid	PLLA discs	Osteoblasts	Collagen increased the substrate’s biocompatibility, enhancing cell survival, cell proliferation, and ALP expression.	Zhao et al. (2014)
	Synthesized 3D structures consist of four PLA membrane surfaces seeded with either MSCs alone or with a co-culture of MSCs and EPCs.	3D printed PLA membranes	MSCs	As a result of the coatings, cells were distributed uniformly across the scaffold, and differentiation of osteoblasts could be observed.	Guduric et al. (2017)
	PEI/PAA/PEI/nanoclay	open-cell polyurethane foam	MSCs	Coated foams have tunable physical (porosity and density) and mechanical (compressive stiffness and strength) attributes. Biocompatible crosslinked coatings for MSCs	Ziminska et al. (2019)
	Chitosan/sodium hyaluronate	hydroxyapatite–gelatin-based 3D-printed scaffolds	MC3T3-E1	The LbL-assembled coating’s application resulted in better mechanical, swelling, and degradation properties. Scaffolds produced an ideal environment for MC3T3-E1 cell adhesion as well as growth.	Chen et al. (2019)
Plasma Treatment	Chitosan-PEO-Coral scaffold modified by Oxygen and Nitrogen plasma treatment		MC3T3 osteoblast	Increased hydrophilicity, which encourages cell adhesion, proliferation, as well as enhanced cell growth	Tabaei et al. (2021)
	PCL modified by Acrylic acid and oxygen		MC3T3-E1	Increased hydrophilicity, enhanced cellular differentiation as well as the proliferation	Ko et al. (2015)

## 9 Tests applied to “composite matrix and multifunctional polymeric scaffolds” for the determination of bioactivity

Bioactivity is the degree to which a material can influence its biological environment. In recent years, researchers have begun to explore the potential of 3D scaffolds for tissue regeneration. They have developed a number of applications for these scaffolds, with researchers hypothesising that scaffolds could potentially provide structural stability and an environment for cellular regeneration, mimicking the functionality of natural tissue. Since then, 3D scaffolds have been tested for a variety of applications, including bone regeneration, nerve regeneration, muscle regeneration, tendon and ligament regeneration and many others. In order to determine the bioactivity of such multifunctional 3D scaffolds and composites *in-vitro* bioactivity and *in-vivo* bioactivity of the scaffolds are assessed.

Presently only two popular techniques are being utilised to evaluate the *in-vitro* bioactivity of bioceramics. One approach is to assess the bioceramics’ capacity to generate apatite in the simulated bodily fluids (Kokubo and Takadama, 2006). This can be performed *via* X-Ray diffraction method and using the ICP-OES technique (Pham Minh et al., 2013). Investigating how *in-vitro* bone cells react to bioceramics is the second alternative method for *in-vitro* bioactivity assessment (Valerio et al., 2004; Sun et al., 2006; Wu et al., 2008). Singh and colleagues developed a scaffold to look at the apatite formation when placed inside SBF in order to assess the apatite formation (Singh et al., 2020b). Prior to doing *in-vivo* bone bioactivity investigations, this technique is helpful and can greatly minimise the number of animals required for *in-vivo* evaluation. The scientific world as a whole has accepted the concept that *in-vitro* cell testing can be used to examine the *in-vitro* bioactivity of bioceramics. This method is known as the cell experiment method (Wu and Xiao, 2009b). This technique is frequently used to evaluate the bioactivity of bioceramics. Numerous instances, however, suggest that employing cell tests to assess the *in-vitro* bioactivity of bioceramics is insufficient (Webster et al., 1999; Manicone et al., 2007a; Manicone et al., 2007b; Popat et al., 2007). Therefore *in-vivo* bioactivity of the biomaterials is determined within the animal model. Three different steps are adopted for the determination of *in-vivo* bioactivity which includes blood sample collection for further biochemical analysis after third and sixth week. Followed by histological preparation in which the newly developed bone is isolated surgically and hematoxylin and eosin staining are performed for examination under a light microscope. Lastly, the biochemical analysis of the isolated serum is performed to determine the total alkaline phosphatase activity and test the other biomarkers like osteocalcin (Al-Rashidy et al., 2020). All the above tests are widely applied by researchers in the field of bone tissue engineering to determine the bioactivity of developed biopolymeric scaffolds.

## 10 Multifunctionality of polymeric scaffolds

Fabrication of biomimetic biomaterials with high porosity and specific morphology is of great interest to the scientific community as it can lead to improved properties. The biomimetic approach is a novel concept in materials science that can tailor material properties such as mechanical strength and stiffness while maintaining beneficial biological functions. Functionalisation of polymeric scaffolds is a common method to make them more suitable for cell growth. While there are many different techniques, most functionalisation methods depend on the material being used and some of these techniques are more effective than others. For example, surface modification method that is widely applied to incorporate the molecules necessary for cellular proliferation. It is done using various polymeric materials to form the desirable structural 3D scaffold. Copolymerisation with the same monomers has been used to functionalise polymer scaffolds in terms of promoting cellular adhesion in order to overcome the parent polymer’s limitations. Fabrication technique also plays an essential role in the functionality of the scaffolds like 3D printing, electrospinning, blowspinning, etc. They help in the development of unique morphologies of microparticles and hydrogels that can possess different mechanical properties, degradation rates and cellular adhesion. Relatively newer techniques for making 3D scaffolds include decellularization and 3D printing methods. Decellularization is a method of producing and functionalising natural 3D scaffolds by extracting an organ from an animal, removing all cells using detergents, and then reimplanting stem cells from a potential host organ. Growth factors can be added to the decellularized scaffold to accelerate cellular differentiation. More recently, bioactive scaffolds have been developed for tissue regeneration through the use of 3D printing. Several organs have been revitalised and cellularized using donor stem cells. A well-known example of this process is a re-cellularized functional heart organ, exhibiting the successful restoration of functionality (Taylor et al., 2020). In addition to the heart, several other organs, such as the lungs (O’Neill et al., 2013) and bladder (Moreno-Manzano et al., 2020), have also been recellularized *in-vitro*. Among these techniques, however, 3D printing has the advantage of enabling precise scaffold dimensions on the nanoscale compared to other traditional manufacturing methods. Countless bioactive materials have been 3D printed into scaffolds over the years. Even hydrogels have been 3D printed to create specialised 3D scaffolds. In this context, biofunctional polymeric materials with significant mechanical strength can serve as a support matrix for cell proliferation, adhesion and osteogenic differentiation with embedded materials for bone tissue regeneration. Table 6 discusses the recent research on the development of multifunctional polymeric scaffolds along with their applications.

**TABLE 6:** Multifunctional polymeric scaffolds with their properties and applications.

Multifunctional scaffolds	Properties	Technique	Applications	References
1. PD-RGO doped PLA	Customizable and mechanically tough, antioxidant, antibiofilm and pro-angiogenic multifunctionality, osteoinductive, biocompatible	3D printing	Bone tissue engineering	Sharma et al. (2022b)
2. Superparamagnetic iron oxide nanoparticles doped Polycaprolactone/Hydroxyapatite	High healing efficiency	3D printing	Bone Tissue engineering	Petretta et al. (2021)
3. Poly lactic-co-glycolic acid/tricalcium phosphate scaffolds containing icaritin	Stimulate both osteogenesis and angiogenesis, enhanced ALP activity and increased osteogenic marker expression, increase bone formation and vascularization *in-vivo*	Low-temperature deposition manufacturing	Bone tissue defects under steroid associated osteonecrosis	Wang et al. (2013b)
4. Poly(D,L-lactic acid) nanofibers coupled with recombinant human bone morphogenetic protein/calcium phosphate particle/poly(lactic-*co*-glycolic acid) nanocomposite fibers	Core–shell structure developed, rhBMP-2 encapsulated in the water phase core of fibers, rhBMP-2 controlled release, balanced osteoinductivity and osteoconductivity, controlled degradation	Dual-source dual-power electrospinning	Bone tissue engineering	Wang and Wang, (2012)
5. Polycaprolactone/gelatin nanofiber films/hitosan/poly (γ-glutamic acid)/hydroxyapatite (CPH) hydrogels/platelet-rich fibrin	Enhanced osteoinduction, osteoconduction, and osseointegration	Electrospinning and lyophilization	Bone tissue engineering	Zhang et al. (2019b)
6. Zinc and reduced graphene oxide. Arabinoxylan, the nanosystem (Zn@rGO), and nanohydroxyapatite polymeric nanocomposites ARX-*g*-(Zn@rGO)/HAp	Antimicrobial activities, increased biodegradation and swelling in PBS, enhanced cell viability and proliferation against preosteoblast cell line	Hydrothermal method/Freeze drying	Bone tissue engineering	Khan et al. (2022)
7. Ultrasuperparamagnetic iron oxide doped Silk fibroin/hydroxyapatite	good porosity, mechanical property, thermal stability for bone repair, osteogenesis enhanced in *in-vivo* system also.	Freeze drying method	Bone tissue engineering	Liu et al. (2020)
8. SiO_2_ incorporated Polycaprolactone, poly(3-hydroxybutyrate)PHB, and PHB doped with the conductive polyaniline	Prolonged drug release provided by dense silica shell	Electrospinning	Drug delivery for bone tissue regeneration	Karpov et al. (2020)

## 11 Doping and its advantages

**FIGURE 7:**
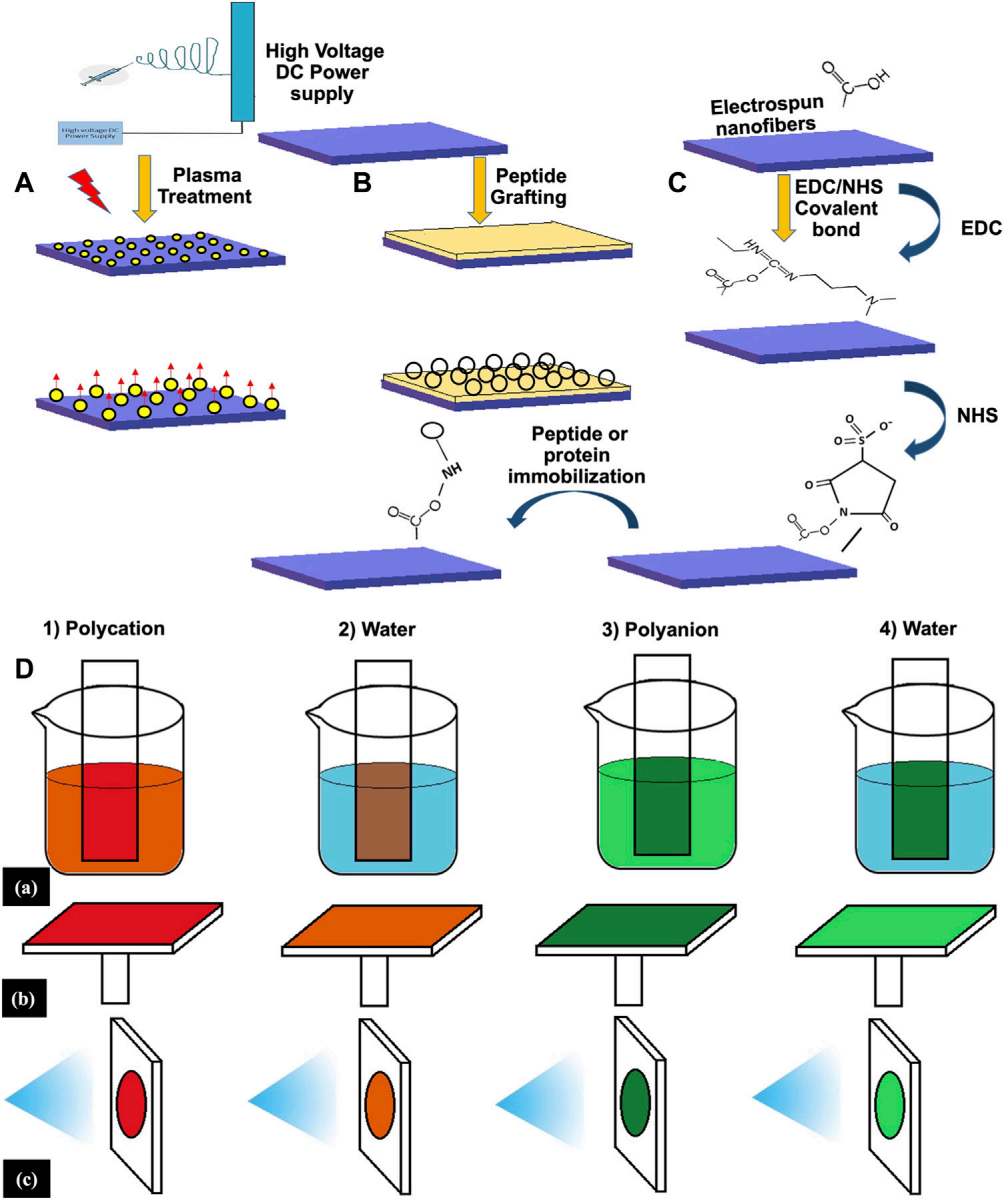
Different doping techniques of scaffold surface: **(A)** Plasma treatment; **(B)**; Peptide Grafting; **(C)** EDC-NHS Coupling; **(D)** Layer-by-layer assembly.

Biomedical sciences (medicine and biology) and materials science have collaborated in recent years to create biomaterials for application in tissue reconstruction and replacement. For this phenomenon, biomaterials need to be physically and chemically stable and possess certain mechanical qualities. Various bio-ceramic materials and BGs are among the most commonly employed materials for bone tissue healing and prosthesis concealing due to their biocompatibility, biodegradability, bioactivity, and osteoconductivity (Cai et al., 2019). nBGs are some of the most thoroughly researched nanomaterials for hard tissue reconstruction and replacement (Philippart et al., 2017). These include calcium phosphates (e.g., hydroxyapatite, tricalcium phosphate and calcium silicates e.g., wollastonite (CaSiO_3_) and larnite (-Ca_2_O_4_). Bone-bonding materials can establish stable chemical contact with the surrounding structures by generating apatite, similar to bone, on the surface. Apatite production *in-vitro*, when exposed to an aqueous media with a chemical composition that mimics physiological liquids, can be used as a predictor of apatite formation *in-vivo*. The bioceramic materials and nBGs of current generations have indeed been developed because of their capacity to induce bone regeneration and resemble biological tissue, eliciting a response from the body similar to that seen in the presence of natural tissue. Materials that can both anchor the implant in place and attract the cells that regulate their breakdown rate have become increasingly important. Bone-forming capability can be achieved by manipulating the release of certain ions during dissolution *in-vivo*. Because of the elevated solubilization rate, the released ions are swayed from the transplantation site by body fluid, which may interrupt the bone tissue healing process during new bone ingrowth. Also, the degradation kinetic growth factors presented by calcium phosphate ceramics are typically unsatisfactory, and the materials lack intrinsic osteoinductivity. The low mechanical strength and fracture toughness of BGs also makes it challenging to implant them in weight-bearing areas (Baino, 2018). Figure 7 displays the various doping techniques like EDC-NHS coupling, layer-by-layer assembly, plasma treatment, etc.

The addition of a trace amount of another element into the bulk material is known as doping. It has been found that an increase in the concentration of dopant ions plays a crucial role in facilitating the formation of new crystalline phases. This means that the conditions in which the thermal treatment is carried out can affect pristine material’s qualities (Vahabzadeh and Bose, 2017; Sikder et al., 2020). Bioceramics and BGs are now integrated with certain metal ions that possess therapeutic benefits like Sr_2+,_ Cu_2+,_ and Zn_2+_ into their chemical structure to mitigate the drawbacks of virgin materials and promote their utilization in a variety of biomedical settings. Past research has shown that minerals, including cations Mg_2+,_ Cu_2+,_ Zn_2+,_ Sr_2+,_ Fe_3+_/Fe_2+,_ Co_2+/3+,_ and Mn_2+,_ are also essential for bone growth, development, and maintenance. Therefore incorporating them into biomaterials has been shown to increase angiogenesis and osteogenesis (Ryan et al., 2019; Yuan et al., 2019; Zhuang et al., 2019). Considering the importance of cations in governing metabolic pathways, doping with Ag_+_ and Cu_2+_ in the structure of bioceramics and nBGs has been extensively researched and shielded against microbial infections in a wide range of biological contexts (El-Rashidy et al., 2018; Predoi et al., 2019; Ryan et al., 2019; Singh et al., 2020c; Hui et al., 2020; Sikder et al., 2020). Some researchers have taken a different track, looking at these materials as drug delivery systems. Targeted nano-carriers or scaffolding components, such as doped-bioceramics, are successfully functionalized with the drug or bioactive molecules to achieve this goal (Morsi and Abd Elhamid, 2019; Singh et al., 2020c).

Researchers investigated the use of rare Earth metals as dopants in bioceramics and nBGs, resulting in the creation of a novel good potential class of luminescent optical nanostructured materials. These materials could substitute organic fluorophores and quantum dots in bio-imaging application fields that range from cancer diagnosis to intra-operative supervision and postsurgical assessment (Neacsu et al., 2019). However, some challenges must be overcome before suitable biocompatibility and biodegradability nanoparticle-imaging sensors can be employed in healthcare situations. Different crystallinity, shape, and stoichiometry in the end products of metal-doped bioceramics and BGs have been achieved by the use of several synthesis techniques (Srinivasan et al., 2019). Methods such as sol-gel (El-Rashidy et al., 2018; Predoi et al., 2019; Zhuang et al., 2019), chemical precipitation (Luo et al., 2018; Gallo et al., 2019; Srinivasan et al., 2019; Chen et al., 2022), the melt-quenching approach (Deng et al., 2019), magnetron sputtering (Vranceanu et al., 2019), and pulsed laser deposition (Duta et al., 2019) are only a few of the many options. Once bioceramics and BGs have proven to be acceptable as coating materials (Predoi et al., 2019; Vranceanu et al., 2019), cements (Li et al., 2018; Zhang and Williams, 2019), scaffolds (Zhao et al., 2014; Yin et al., 2018; Luo et al., 2019; Yuan et al., 2019; Zhuang et al., 2019), and nanoparticles (El-Rashidy et al., 2018; Chen et al., 2019; Morsi and Abd Elhamid, 2019; Singh et al., 2020c), the optimum synthesis process will depend on the application. Over the past decade, researchers have analyzed the literature to determine the effects of metal ions in various inorganic matrices for application in a wide variety of diseases. About metal ion doping of inorganic matrices, they describe, summarise, and evaluate. Several studies using ion-doped inorganic matrices for bone tissue engineering have risen in the past years, which shows the growing interest in these materials among scientists. As a result, silicon, sodium, and phosphorus, the primary chemical components of silicate-based BGs and bioceramics, have attracted a major group of scientists and clinicians. Physicochemical, structural, and biological behaviour within *in-vitro* and *in-vivo* environment are discussed in this article showing the effectiveness of ions like magnesium, silver, copper, strontium, lithium, and cobalt. Table 7 discusses the latest research done on the doping of materials and their application in BTE.

**TABLE 7:** Doping and its advantages in BTE.

Scaffold material	Dopant	Positive effect on bone tissue regeneration	References
Single-doped scaffolds			
Hydroxyapatite	La	La3^+^ stimulated macrophage proliferation and activity by activating the Wnt/-catenin signaling pathway, improving osteogenic proliferation and differentiation. La-HA/CS scaffolds demonstrated osteoinduction as well as biodegradation capabilities.	Yin et al. (2019)
Mesoporous calcium silicate		Gradual deterioration of scaffolds released La^3+^ ions stimulating the TGF-β signaling pathway, which in turn encouraged the rBMSC proliferation along with osteogenic differentiation.	Peng et al. (2019)
Whitlockite	Ce	The inclusion of Ce^3+^ to whitlockite decreased its crystallinity, activating the SMAD signalling system, increasing osteogenic activity, upregulating the expression of the osteogenic genes, and accelerating bone repair.	Hu et al. (2019)
Mesoporous calcium silicate	Eu	The luminescent Eu-mesoporous calcium silicate scaffolds could be utilized to mark and identify *in- vitro* cultured cells and nascent bone growth *in-vivo*. Eu^3+^ can promote bone regrowth as well as enhance osteoporotic development.	Wu et al. (2016)
Bioglass	Gd	Through the Akt/GSK3beta mechanism, Gd^3+^ increased the development of hBMSCs and accelerated the process of bone induction.	Zhu et al. (2019)
Bioglass	Ho	Reduced rapid cation leaching as well as stabilized dissolution of glass	Delpino et al. (2021)
Co-doped scaffolds			
Hydroxyapatite	Yb/Er	When exposed to a 980 nm infrared laser, Yb/Er-hydroxyapatite nanorods displayed significant fluorescence intensity as well as light stability, were able to track the site of the BMP-2 protein translocation, as well as were biocompatible and capable of osteogenesis.	Liu et al. (2019)
	Sm/Eu	It encourages hASC growth and possesses luminous properties.	Alicka et al. (2019)
	La/Pr	Compared to single and stoichiometric hydroxyapatite, co-substitutions showed higher bioactivity, better cell viability, and higher antibacterial efficacy.	Chandran and Am, (2021)
Fluorapatite	Yb/Ho	The doping ratio of Yb3+ and Ho3+ was changed to increase the luminous efficacy of up-conversion. Dextran-modified water-soluble fluorapatite nanoparticles were also used for biological imaging and cell labelling.	Li and Chen, (2016)

## 12 Future perspective

There have been significant efforts to replace, image, or regenerate bone using cutting-edge nanotechnology as a consequence of a developing understanding of how bone tissues’ nanoscale characteristics contribute to their distinctive capabilities. Novel discoveries in the understanding and engineering of bone tissue are the consequence of these efforts. Several challenges still exist, though.

Creating vascular and innervated bone tissue is among the most challenging problems that current nanofabrication techniques have yet to solve. Nanoparticles and their technologies must be integrated with upgraded materials and processes. Manufacturing methods like multi-material bioprinting should be used in order to imitate the multiple-scale “organized chaos” found in bone tissues, including the cellular hierarchy and the ECM. Such pairings run into difficulties because of the mechanical differences between hydrogels, which are often used in bioprinting, and relatively hard materials ideal for bone ECM modelling.

It is predicted that the development of innovative bioinks and high-resolution printing methods would significantly impact future breakthroughs in hierarchical osseous tissue bioprinting. The advent of medicine will increasingly focus on the patient and be more customized; it is already commonly accepted. To employ powerful algorithms to influence advertising and subsequent purchase decisions, online firms already collect large volumes of data about consumer populations; nevertheless, assessments of medical treatment are based on surprisingly little data. Combining breakthroughs in nanotechnology with fields like big data research, genomics, and proteomics, among others, may have unanticipated implications for the TE sector.

In immunology, for instance, a patient’s particular biology cannot be avoided, yet illnesses, not individuals, are the focus of small molecule medication development. An initial move toward individualized care is the utilization of patient-explicit cells for bone TE, like MSCs and, at this point, unstudied iPSCs. Bone TE strategies will get redone as additional patient information is received and dissected at lower costs.

It may also be necessary to use pico-technology or engineering at sizes smaller than 10^−9^ m by changing the electrical environment of atoms and molecules to figure out how to arrange collagen and mineral phases to replicate the intricate natural structure of bone. Recent evidence suggests that the shape and handedness of calcium carbonate crystals may be changed by the addition of chiral amino acids, much like the helical forms that naturally occur in bone.

The insertion of small molecules with varying electron distributions is an early form of constructing nanostructures from “below.” The ability to customize medications and use pico-technology will undoubtedly improve the adequacy of nanomedicine and the likely harmfulness of nanomaterials. For example, NPs’ organic personality and potential as medication conveyance stages will be emphatically affected by the normally happening protein crown that encompasses them. NPs that advance the adsorption of supplement proteins will be obliterated before arriving at the designated tissues, as opposed to NPs that have some level of secrecy abilities in view of their surface properties, like electron dispersion.

The most recent finding that is; patient and illness type-dependent variations in NP corona composition exist emphasizes the complexity that nanomedicine researchers are being forced to accept more and more. The availability of additional patient-specific data and a greater comprehension of NP properties at the smallest scales will influence future nanomedicine initiatives. Finally, it has been demonstrated that decellularized bone matrices are more appealing than bottom-up scaffold design. This is because, despite their best efforts, TE scaffolds for bone have yet to imitate the unique physical properties of bone that derive from its multiscale architecture.

It will be important to acquire a more profound comprehension of the scattering of nanoparticles in a solid stage, and the interfacial connections between the two gradually ease that leading to drive transmission to make tissues that can imitate the innate durability and strength of bone from the base (nanoscale) up, potentially with the guide of pico-technology, refined virtual experiences, and information science.

Enhanced assessment procedures are needed to verify the suitability and effectiveness of nanoengineered systems for bone engineering. The possibility of therapies working *in-vivo* will undoubtedly rise with more accurate testing under *in-vivo* settings, including interstitial flow, various cell types organized in three dimensions, and mixtures of bodily fluids, including growth factors and serum proteins.

Additive manufacturing based techniques, notably three-dimensional printing and bioprinting, demonstrated tremendous promise in applications involving tissue engineering owing to their ability to customise (including both temporal and spatial control) in the fabrication of grafts or structures made from tissue that is specific to the patient. By leveraging tissue-specific printed structures made with a range of AM-based technologies, both *in-vivo* and *in-vitro* research has made major advancements in the regeneration of hard tissues.

Despite several research initiatives to improve the desirable properties of printable structures made of HAp-based substances, the conception, development, and treatment of HAp-based bioinks to obtain desired properties, notably for implants that bear load, in accordance with a particularly tough type of tissue and/or organ to achieve desirable qualities in the native hard tissue environment remains a substantial obstacle. Proper bioink design and development are necessary to enable tissue regeneration resembling natural tissue with bioprinted implants (i.e., implant integration, progressive remodelling, development and vascularization). This entails considering concerns linked to printing in terms of the components, cells, processing configurations (pre/post printing), and so forth.

This necessitates a deep grasp of biomaterials, cells, techniques of printing, and the *in-vivo* biological milieu. Throughout the printing process, additional significant obstacles include the possibility of nozzle obstruction and the creation of mechanically stable designs. In addition, printing more advanced 3D designs that make use of a variety of materials, cell types, and printing procedures to produce cellular diversity and functionality *in-vivo* is a significant challenge.

In the field of tissue engineering, it is also extremely difficult to bring 3D-printed biomaterials into the clinic. To investigate structural, chemical, physicomechanical, rheological, biological, and immunological issues using a multidisciplinary approach, significant and crucial tissue engineering research efforts are required.

## 13 Conclusion

This review illustrates the interface research on the physiology of bone and the various bone repair strategies for restoring and replacing damaged or defective bone. All the results that have been investigated in recent studies suggested useful information for creating new biomaterial-based products. However, it is imperative to look at the outcomes of each research trial. Bone graft replacements, implantable materials and scaffolds, tailored 3D structures, and preferred surface qualities are only a few of the several bone restoration techniques. These techniques are inclusive of the factors involved in the development of such biomaterials that can restore and repair damaged bone tissue. Like surface properties of the scaffold material that has a major role in cell adhesion and tissue formation. This article gives insight into how surface modification can enhance bone tissue regeneration. Similarly, pore size and pore morphology which have an adequate response towards cell adhesion and proliferation can also be modified by altering the material choice and fabrication method. It has been observed in past studies that as the size of the bone lesions increases, the role of the bioactive components enhances. Therefore these days, at the pre-clinical stage, signalling factors, polypeptides, and small biomolecules are evaluated for this purpose. By combining these bioactive compounds with cutting-edge carriers, it may be possible to build a delivery system (scaffold) that delivers the active molecules in a consistent and beneficial quantity. However, cell-based methods can also be used to treat significant and complex bone abnormalities, more controlling phases are necessary for future advancements in order to ensure effective clinical treatment.

## Glossary

TE: Tissue engineering
BTE: Bone Tissue engineering
MSCs: Mesenchymal stem cells
ECM: Extracellular matrix
HAp: Hydroxyapatite
TCP: Tricalcium phosphate
3D: Three dimensional
PLA: Poly lactic acid
PVA: Polyvinyl alcohol
PGA: Polyglycolic acid
PMMA: Polymer of acrylamide
Dex-U: Urethacrylate dextran **PLLA** Poly(L-lactic acid)
PADH: Polymer of acrylamide -Urethacrylate dextran
BG: Bioactive glass
HNTs: Halloysite nanotubes
PMPV: Polyacrylamide/poly (vinyl alcohol)
AM: Additive manufacturing
PVA/SA/HA: Polyvinyl alcohol/sodium alginate/hydroxyapatite
MGO: Magnetic graphene oxide
GAG: Glycosaminoglycan
SLRPs: Small leucine-rich proteoglycans
TNF-alpha: Tumor necrosis factor-alpha
BMPs: Bone morphogenetic proteins
VEGF: Vascular endothelial growth factor
Col: Collagen
Gel: Gelatin
Ch: Chitosan
HA: Hyaluronic acid
PD-RGO: Polydopamine-reduced graphene oxide
SF: Silk fibroin
AA: Amino acid
CS: Chondroitin sulfate
Alg: Alginate
SBF: Simulated bodily fluids
rhBMP-2: Recombinant human bone morphogenetic protein
EDC: 1-Ethyl-3-(3-dimethylaminopropyl)carbodiimide
NHS: N-hydroxysuccinimide
CL: Cellulose
PLGA: Poly (lactic-co-glycolic acid)
GFs: Growth factors
PEG: Polyethylene glycol
PCL: Poly ɛ-caprolactone
ICP-OES: Inductively coupled plasma-optical emission spectrometry
HP: Hyperbranched polymer
PU: Polyurethane
PPF: Poly(propylene fumarate)
QDs: Quantum dots
rBMSC: Recombinant Bone marrow stromal cells
SMAD: Mothers against decapentaplegic
hASC: Human adult stromal cells
SBMs: Smart biomaterials
PVDF: Polyvinylidene fluoride
SAMs: Self assembled monolayers
CNTs: Carbon nanotubes
f-MWCNT: Multiwalled Carbon Nanotubes
ERMs: Enzyme-responsive materials
